# Qualitative and quantitative evaluation of thylakoid complexes separated by Blue Native PAGE

**DOI:** 10.1186/s13007-022-00858-2

**Published:** 2022-03-03

**Authors:** Éva Sárvári, Gabriella Gellén, Máté Sági-Kazár, Gitta Schlosser, Katalin Solymosi, Ádám Solti

**Affiliations:** 1 Department of Plant Physiology and Molecular Plant Biology, Institute of Biology, Faculty of Science, ELTE Eötvös Loránd University, Pázmány Péter sétány 1/C, Budapest, 1117 Hungary; 2 MTA-ELTE Lendület Ion Mobility Mass Spectrometry Research Group, Institute of Chemistry, Faculty of Science, ELTE Eötvös Loránd University, Pázmány Péter sétány 1/A, Budapest, 1117 Hungary; 3 Doctoral School of Biology, Institute of Biology, Faculty of Science, ELTE Eötvös Loránd University, Pázmány Péter sétány 1/C, Budapest, 1117 Hungary; 4 Department of Plant Anatomy, Institute of Biology, Faculty of Science, ELTE Eötvös Loránd University, Pázmány Péter sétány 1/C, Budapest, 1117 Hungary

**Keywords:** Bundle sheath, Blue/Clear Native PAGE, Chloroplast NADH dehydrogenase-like complex, Lincomycin, Maize, Mesophyll, Photosystem I, *Populus*

## Abstract

**Background:**

Blue Native polyacrylamide gel electrophoresis (BN PAGE) followed by denaturing PAGE is a widely used, convenient and time efficient method to separate thylakoid complexes and study their composition, abundance, and interactions. Previous analyses unravelled multiple monomeric and dimeric/oligomeric thylakoid complexes but, in certain cases, the separation of complexes was not proper. Particularly, the resolution of super- and megacomplexes, which provides important information on functional interactions, still remained challenging.

**Results:**

Using a detergent mixture of 1% (w/V) *n*-dodecyl-*β*-d-maltoside plus 1% (w/V) digitonin for solubilisation and 4.3–8% gel gradients for separation as methodological improvements in BN PAGE, several large photosystem (PS) I containing bands were detected. According to BN(/BN)/SDS PAGE and mass spectrometry analyses, these PSI bands proved to be PSI-NADH dehydrogenase-like megacomplexes more discernible in maize bundle sheath thylakoids, and PSI complexes with different light-harvesting complex (LHC) complements (PSI-LHCII, PSI-LHCII*) more abundant in mesophyll thylakoids of lincomycin treated maize. For quantitative determination of the complexes and their comparison across taxa and physiological conditions, sample volumes applicable to the gel, correct baseline determination of the densitograms, evaluation methods to resolve complexes running together, calculation of their absolute/relative amounts and distribution among their different forms are proposed.

**Conclusions:**

Here we report our experience in Blue/Clear-Native polyacrylamide gel electrophoretic separation of thylakoid complexes, their identification, quantitative determination and comparison in different samples. The applied conditions represent a powerful methodology for the analysis of thylakoid mega- and supercomplexes.

**Supplementary Information:**

The online version contains supplementary material available at 10.1186/s13007-022-00858-2.

## Background

In oxygenic photosynthesis, the conversion of the energy of sunlight into chemical energy is carried out by the multi-subunit complexes of photosynthetic membranes. All these complexes, the photosystem (PS) I and PSII with their light-harvesting complexes (Lhc/LHC), the cytochrome (Cyt) *b*_*6*_*f* complex, chloroplast NADH dehydrogenase-like complex (NDH), and ATP synthase (ATPs), are composed of numerous polypeptides many of which are highly hydrophobic [1, 2]. To understand the regulation of photosynthesis, it is important to follow qualitative and quantitative changes in thylakoid composition under different circumstances. To this purpose, these components can be examined by different denaturing and native polyacrylamide gel electrophoresis (PAGE) methods. Two-dimensional PAGE using isoelectric focusing in the first dimension and sodium dodecyl sulphate (SDS) PAGE in the second dimension, separating the proteins by both charge and size, gives high resolution. However, it is often difficult to rehydrate and solubilise delipidated hydrophobic membrane proteins, thus it cannot be easily used for the investigation of changes in the quantitative aspects of thylakoid proteome [3]. Native separation methods are, indeed, more convenient. Green PAGE techniques [4–8] apply non-denaturing non-ionic detergents for solubilisation but proper resolution cannot be achieved without the presence of a small amount of ionic detergent that gives charge to hydrophobic proteins. Nevertheless, mega- and supercomplexes of thylakoids as well as association of LHCII antennae are very sensitive to the presence of most ionic detergents that generally leads to the loss of multiple complex interactions. In addition, even sophisticated and mild gradient centrifugation [9, 10] does not allow the separation of intact complexes with high resolution in one step. At the same time, Blue Native (BN) PAGE introduced by Schägger and von Jagow [11] applied in both one- and two-dimensional separations gives superior quality of resolution of membrane complexes, the supramolecular organisation of which is well retained [3, 12–17]. In the application of BN PAGE, after gentle solubilisation of membranes, anionic dye Coomassie Brilliant Blue G-250 is added to the samples. It binds, on a non-covalent way, to the surface of both hydrophilic and hydrophobic residues providing a negative charge that enhances migration of complexes towards the anode and hampers aggregation but does not influence the protein–protein interactions. Thus, separation of complexes by BN PAGE allows obtaining structural information about the native state of the thylakoid complexome in a time efficient way in one separation step.

BN PAGE has been widely used to compare thylakoid complexes in different plant species [18, 19], to follow their changes during development [20, 21], acclimation to various environments [22–25], under stress circumstances [26–31], and in mutant plants [32–35]. In some cases, a useful variant of BN PAGE, Clear Native (CN) PAGE, showing similar band pattern to BN PAGE, was also applied [16, 36]. Mega- and supercomplexes are better resolved by large-pore BN-PAGE [16, 17, 22]. Nowadays, complexome profiling, i.e. mass spectrometry (MS) determination of polypeptide content of bands cut out of lanes or obtained by slicing the lanes, offers great possibilities for discovering new bands and new proteins in the band pattern but requires large-scale MS capacity [37].

Previous analyses unravelled multiple monomeric and dimeric/oligomeric thylakoid complexes. The monomers and PSII supercomplexes (PSII-s) are well characterised [9, 17, 38]. PSII-s differing in antenna size are C_2_S_2_M_2_, C_2_S_2_M, C_2_S_2_, C_2_S, where C, S and M are standing for PSII core, strongly (via Lhcb5) and moderately bound (via Lhcb4 and Lhcb6) LHCII trimers building of Lhcb1,2 and Lhcb1,3, respectively. The monomer PSI complex is built of PSI core components and LHCI composed of Lhca pigment-proteins organised in dimers (Lhca1,4 and Lhca2,3), and arranged in a semicircle around the core between PsaG and PsaK [39]. The PSI may also bind additional LHCII trimer on the opposite side of the complex (known as PSI-LHCII complex), and this LHCII is highly efficient in light harvesting for PSI and thus important in excitation energy allocation during light acclimation process [10, 39–41]. More LHCII trimers connected to PSI were discovered using milder methods for solubilisation [42–45]. The composition of PSI bands larger than PSI-LHCII obtained by BN PAGE, however, is not totally clear, though several megacomplex (mc) and supercomplex bands consisting of only PSI or also containing PSII and/or LHCII were reported in *Arabidopsis* and other higher plant thylakoids [16, 17, 22, 36, 38, 46]. Although PSI-Cyt *b*_*6*_*f* or PSI-NDH complexes involved either in cyclic electron flow or chlororespiration, respectively, were isolated [47] or detected by single particle analysis [43, 48], only PSI-NDH complex could be separated by BN PAGE [16, 37, 49, 50].

The primary aim of this paper is to show the qualitative and quantitative evaluation of BN-/CN PAGE patterns of poplar and maize thylakoid models. As a powerful technique, here we report the way of retaining the native state of mega-/supercomplexes by solubilisation with 1% (w/V) *n*-dodecyl-*β*-d-maltoside (*β*-DM) plus 1% (w/V) digitonin mixture and increasing the resolution of the large complex region of BN PAGE using 4.3–8% gel gradient, which is employed for the detection of several PSI mega/supercomplexes in untreated bundle sheath (BS), and lincomycin (LM) treated mesophyll (M) thylakoids. We demonstrate the quantitative comparison of complexes in untreated and LM treated M thylakoids involving evaluation methods to show how complexes running together can be resolved, and how their absolute/relative amounts and distribution among their different forms can be calculated.

## Results and discussion

To follow the variations of thylakoid composition in different samples and under various conditions, it is necessary to extract the complexes in their native form, separate them as clean bands identified by their polypeptide composition and to assess changes in their quantities.

### Evaluation of gel patterns

As a frequently used system, we applied short gels for BN PAGE [Mini-Protean II Bio-Rad apparatus] as it was advised to save reagents and achieve a faster and milder separation [16]. Long gels are not so beneficial because the bands sometimes are less sharp and the resolution of bands is usually not better than in short gels [27, 51]. However, slightly longer gels than the ones used in Mini-Protean II may be more convenient. In the second or third dimensional SDS PAGE separations of polypeptides we used Laemmli gels [52] with glycerol which gives sharp bands/spots (Additional file 1: Fig. S1b, d) and quite good resolution both in the high and low molecular mass region. However, Tricine-SDS PAGE [53] or SDS gels containing urea [16] are used the most frequently because of their higher resolution in the low molecular mass region, and better separation of Lhc apoproteins. Robust methods should operate across taxon borders. Since starting trials of our BN PAGE investigations were performed on poplar thylakoids, evaluation of gel pattern will be demonstrated on this model.

#### Identification of thylakoid complexes

**Fig. 1:**
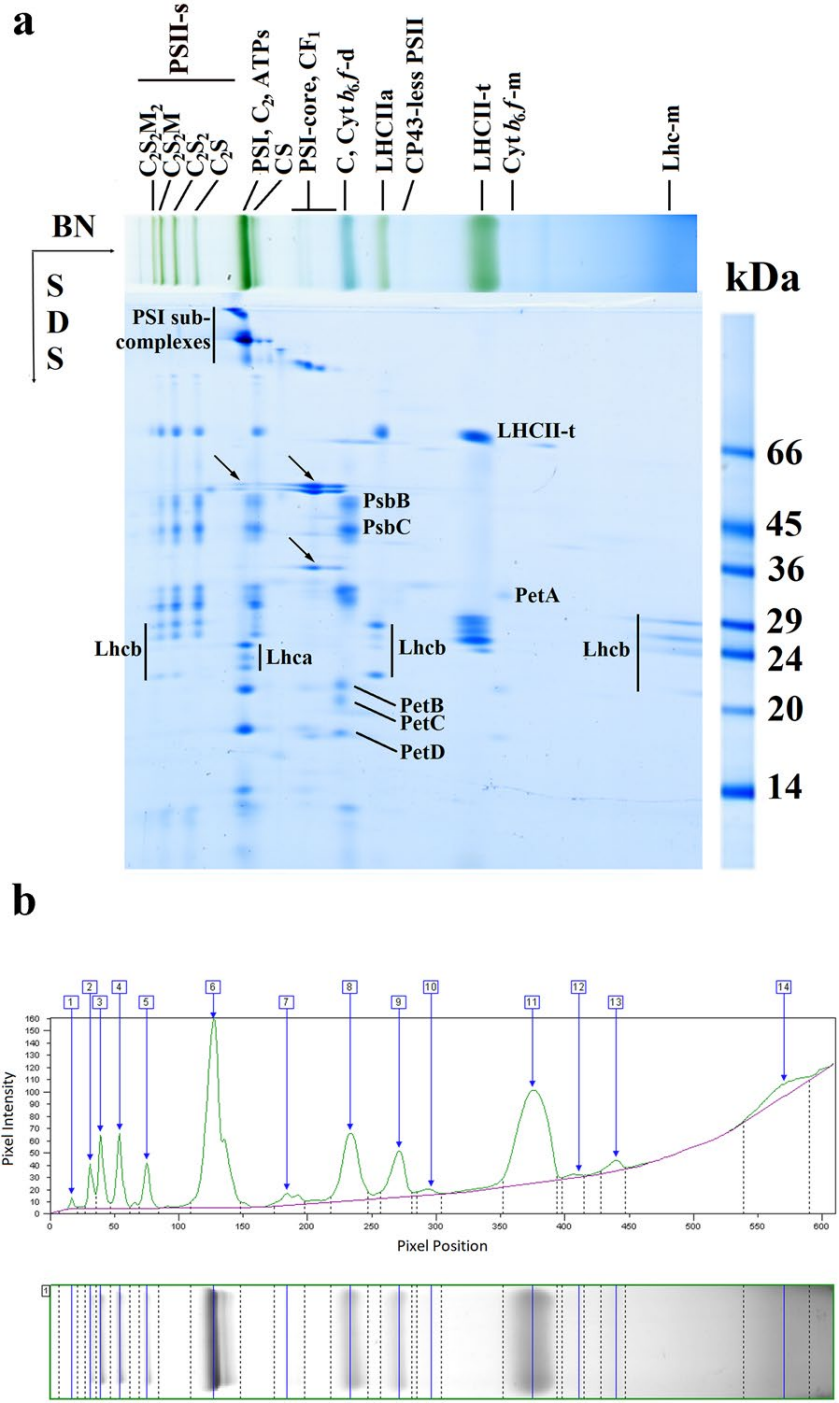
BN/SDS PAGE pattern of poplar (*Populus jacquemontiana* var. *glauca*) thylakoid complexes. **a** Thylakoids (500 µg Chl mL^−1^) were solubilised with 2% (w/V) *β*-DM and separated in 5–12% BN gel gradient followed by SDS PAGE. C, S and M: PSII core, strongly (S) and moderately bound (M) LHCII trimers; CP: chlorophyll-protein; LHCII-a: CP29 + CP24 + LHCII-t; s: supercomplex; t: trimer d: dimer; m: monomer; Cyt: cytochrome; ATPs: ATP synthase; CF_1_: coupling factor 1. Protein standards (kDa) were as follows: bovine serum albumin (66), ovalbumin (45), glyceraldehyde-3-phosphate dehydrogenase (36), carbonic anhydrase (29), trypsinogen (24), trypsin inhibitor (20), *α*-lactalbumin (14). **b** Baseline of the densitogram of poplar thylakoid complexes separated by BN PAGE shown in **a** made by the Phoretix program (adjusted by the user)

Identification of thylakoid complexes are based on their characteristic polypeptide composition (Fig. 1a) determined as in Basa et al. [28]. Otherwise, in angiosperms, complexes separated by BN PAGE were qualitatively similar apart from variations in abundance in the different species (Additional file 1: Fig. S2). PSI core monomers together with LHCI (Lhca1–4) were found in PSI and depending on the detergents used also in PSI-LHCII (binding a trimeric LHCII) bands. PSII dimer (PSII-d: C_2_) showed very similar mobility to PSI. Interestingly, another PSII complex running a little further than PSII-d and containing some LHCII, probably CS [9], was also detectable in poplar. Components of PSII were also present in PSII supercomplexes, PSII monomer (PSII-m: C), and chlorophyll-protein-43 (CP43)-less PSII (only a very faint band in poplar). Free Lhc bands occurred as LHCII assembly (LHCII-a) containing LHCII trimer (LHCII-t) together with CP29 and CP24, LHCII-t, and Lhc-m. LHCI was strongly bound to PSI core, so Lhca was rarely found in the Lhc-m band. The PSI subcomplexes and LHCII-t bands contained some residual bound chlorophyll (Chl) even after the denaturing separation (see for maize in Additional file 1: Fig. S1). Except for a few cases, the Cyt *b*_*6*_*f*-d did not separate well from PSII-m. In the case of stronger solubilisation, a Cyt *b*_*6*_*f*-m band also appeared below the LHCII-t band. Regarding the ATPs complex, most coupling factor 1 (CF_1_) was washed out from the surface of thylakoids [52] to be able to solubilise the thylakoids by lower amounts of detergents. Remaining complexes were present as bands containing ATPs complex around PSI and CF_1_ complex around the PSII monomer band (arrows in Fig. 1a).

#### General aspects of quantification of thylakoid complexes

**Fig. 2:**
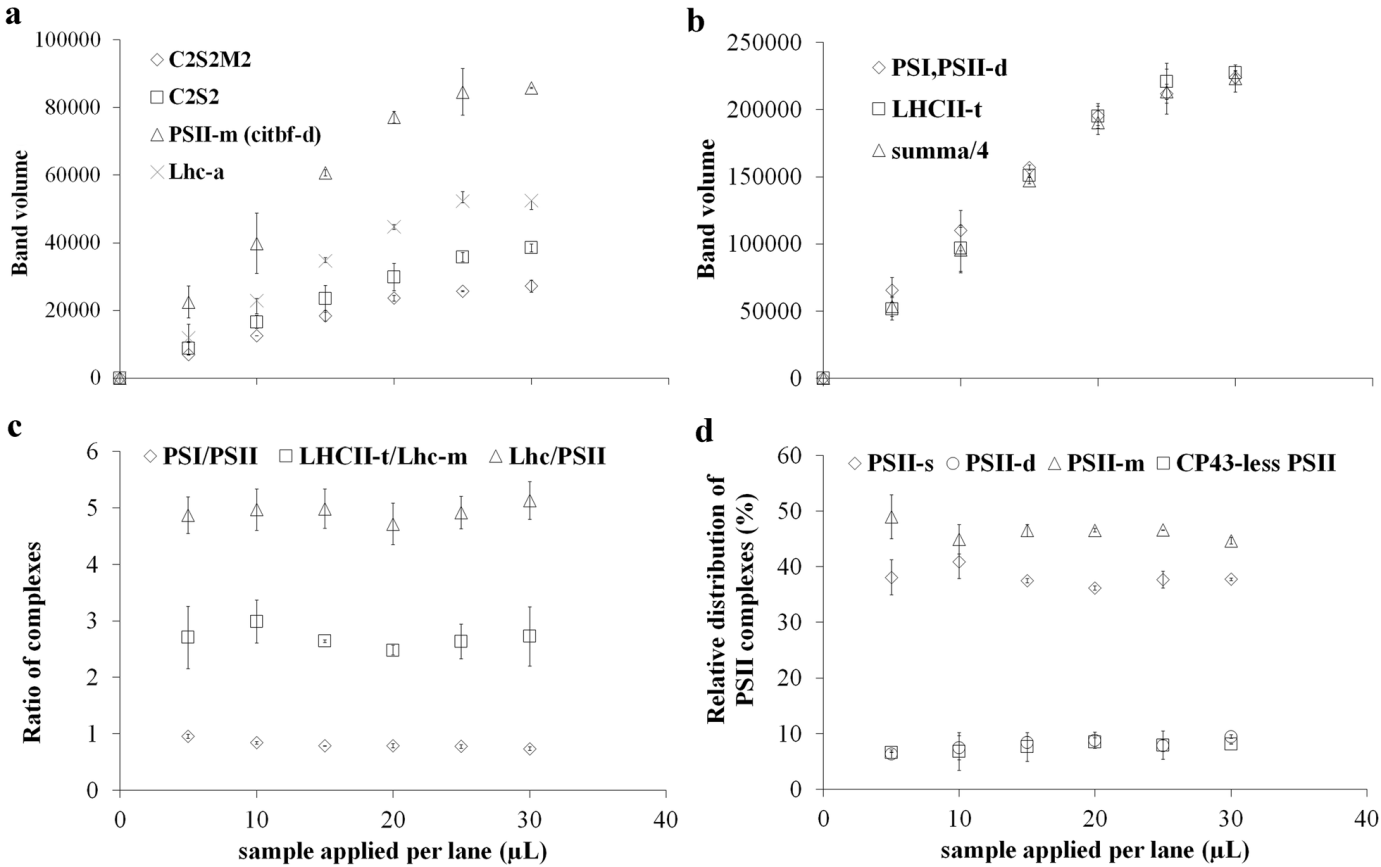
Proportionality of the thylakoid complexes in BN gels. **a**, **b** Band volumes, **c** ratios of complexes, and **d** distribution of the different PSII forms as the function of the sample volume applied per lane. Complexes are marked as in Fig. 1a, s: supercomplex, d: dimer, m: monomer, summa: total band volumes/lane. Poplar thylakoids (500 µg Chl mL^−1^) were solubilised using 2% (w/V) *β*-DM and separated in 5–12% gel gradient with three technical repetitions. PSI/PSII and LHCII-t/Lhc-m ratios were calculated from BN bands, LHCII/PSII and distribution of PSII among its different forms are calculated from SDS gel patterns: all Lhcb spots and PsbB spots were taken into account (see Additional file 2)

Densitometry is used for determination of the amounts of complexes or polypeptide spots. Here we applied the Phoretix program for the quantitative evaluation of BN and SDS gels. Nevertheless, any densitometry applications that enable the quantitative integration of pixel densities on the given band/spot can be also used for evaluation. Comparison of the quantification of bands in Fig. 1a with Phoretix and the open-source ImageJ is given in Additional file 2. To get the real density of bands (calculated as the integrate band volume of the densitogram by the program), the correct determination of the baseline of the densitogram, which is not always a straight line and highly dependent on the structure of the gels, too, is absolutely necessary. Baseline must be adjusted so that those areas of lanes where there are no bands are regarded as zero values (Fig. 1b). Densitometry is based on light absorption by any pigments in the gel that results in dense areas, so we must apply well-defined amounts of Chl (protein) on BN PAGE lanes when Chl-protein complexes are separated. For correct quantitative determinations, band volumes must be directly proportional to the applied amount of protein or Chl. The band volumes and the total volumes per lane [summa] were directly proportional up to 20 µL of 500 µg Chl mL^−1^ sample application, and the ratio of complexes were also invariable (Fig. 2a–c). Important to remark, bands/spots of the same complexes/proteins can only be compared because of the staining variability of different complexes/proteins.

Quantitative determination is more complicated when complexes do not separate well and remain together in the same band. This situation may happen among the numerous mega- and supercomplexes, but mainly in the band where PSI move together with PSII-d, as well as in PSII-m band that also includes Cyt *b*_*6*_*f*-d, and CF_1_ complexes. In certain cases, even the amount of the Lhc-m band cannot be determined perfectly due to a partial overlap with the Coomassie dye front. One can estimate the amount of PSII-d and PSII-m bands comparing the amount of the CP47 apoprotein bands (PsbB) in C_2_ and C to that of C_2_S in the denaturing pattern. This latter gives a well separated band in BN gels. Their ratios in BN gel should be very similar to those determined in the denaturing gel because the polypeptide pattern and pigment content of these complexes are only slightly different, and Coomassie and pigment stains give the pixel density of bands on BN gels. Similarly, the amount of Lhc-m on the BN lanes can be calculated using the ratio of its stained polypeptides either to those of LHCII-t or LHCII-a in the denaturing gel. Estimation of the amount of Cyt *b*_*6*_*f*-d in PSII-m band that also contain CF_1_ is a little more complicated. Once the amount of PSII-m is determined, the remaining volumes in the complex band can be roughly distributed between Cyt *b*_*6*_*f*-d and CF_1_ according to the ratio of their apoprotein amounts detected in denaturing gels.

Another challenge in the quantification of thylakoid complexes is that they represent not only different complexes but also different forms of the same type of complex due to solubilisation but even for such physiological reasons that thylakoids are in different developmental or acclimation stages. From the point of view of membrane dynamics, it is important, indeed, to follow the alterations among these forms. Quantities of complexes which do not show fundamental differences in their composition, e.g. similar PSI complexes or Lhc mono/oligomers, can be estimated from the first-dimensional BN band patterns. However, exact proportion of PSII complexes with different antenna complements cannot be quantified on a similar way. Their proportions can be evaluated from the SDS PAGE pattern using their apoCP47 (PsbB) spot, which is present in all complexes. Basically, it corresponds to the more elegant D1 dot immunoblot method offered by Koochak et al. [54] but can be performed easier. The relative distribution of a complex among its different forms used to characterise changes in the interaction of the different complexes were also invariable in the 5–20 µL range (Fig. 2d). An example of all these calculations is given in Additional file 2: BN-solubilisation, evaluation.

Practical examples of quantitative determinations will be given in chapters characterising large PSI complexes and comparing different thylakoid samples.

### Methodological improvements to separate mega-/supercomplexes in native form

As a second model, maize thylakoids isolated from untreated and LM treated maize seedlings were used to demonstrate the extraction and resolution of thylakoid complexes, particularly PSI mega-/supercomplexes. In maize leaves, the operation of C_4_ type photosynthesis requires the collaboration of M and BS cells containing granal and agranal chloroplasts, respectively [55]. BS thylakoids contain low amount of PSII complexes. Therefore, they are more convenient for studying large PSI complexes. LM is an inhibitor of prokaryotic type translation [56]. Thus, it strongly interferes with protein synthesis in the chloroplast inhibiting the translation of chloroplast encoded core polypeptides of PSI and PSII but does not affect the accumulation and import of the nuclear encoded Lhc-s [57–59]. The high amount of antennae relative to the core complexes may change the complex interactions.

#### β-DM—digitonin detergent mixture gives almost complete solubilisation of maize thylakoids and retain the native state of complexes

To get a realistic picture about the composition of thylakoids it is important to achieve a complete solubilisation but at the same time to retain the native state of the complexes as far as possible. This mainly depends on the type and concentration of detergent used for solubilisation. The *β*-DM and digitonin are the most frequently used detergents in BN PAGE [3, 17]. A digitonin—*β*-DM/α-DM mixture was found to be beneficial in sucrose gradient isolation of PSI-LHCII [10] and getting a more stable C_2_S_2_M_2_ supercomplex [60], respectively. Earlier, with Deriphat PAGE, we also got better results with detergent mixtures than using simple detergents in green gels [7]. Therefore, we investigated the efficiency of mild detergents, digitonin and *β*-DM used in BN PAGE and for comparison *n*-dodecyl-sucrose (DS) used in Deriphat PAGE [7], together with that of *β*-DM/DS—digitonin mixtures for the solubilisation of thylakoids and for the retention of complex interactions.

**Fig. 3:**
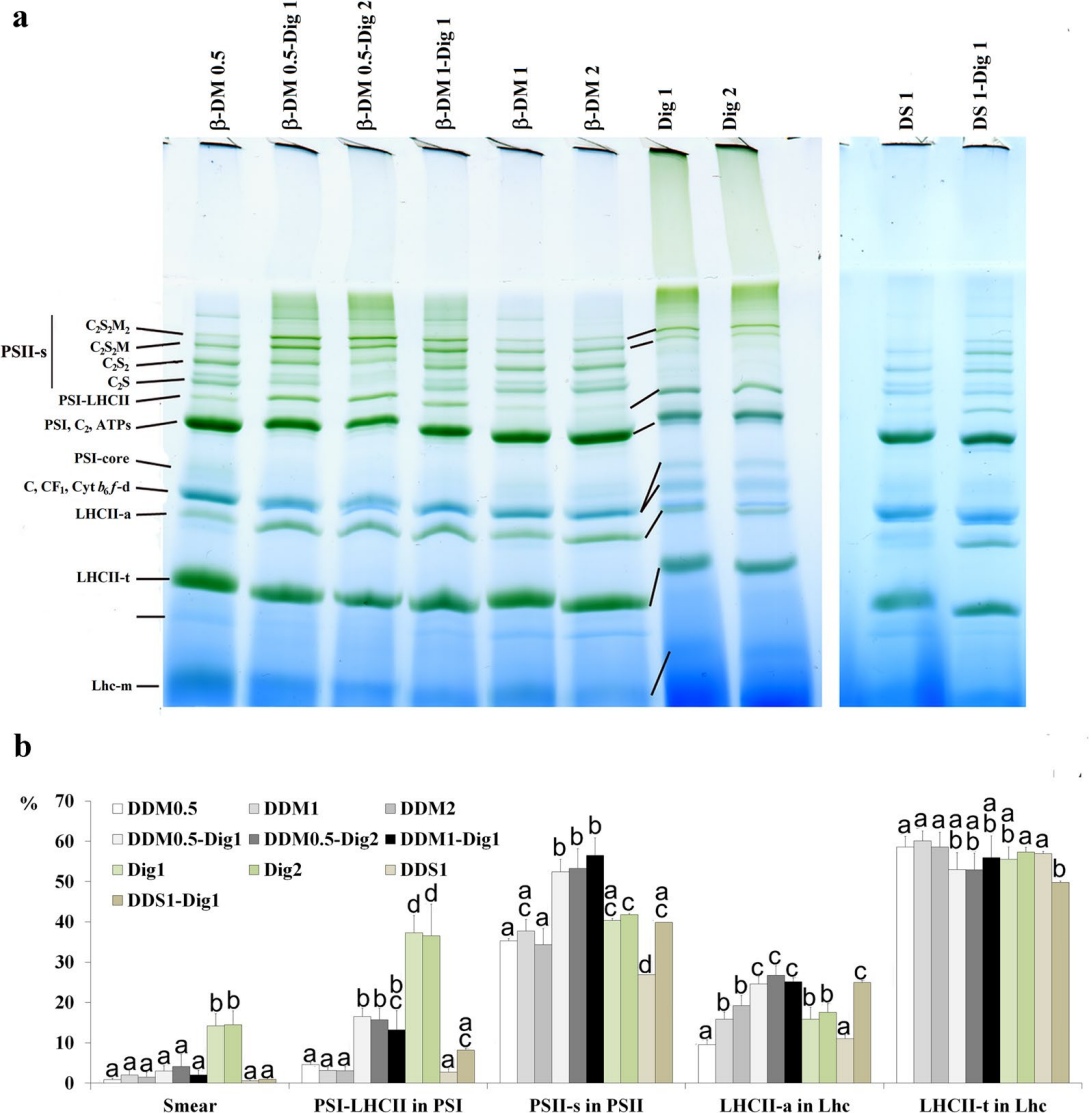
Effect of solubilisation protocols on the complex pattern of maize mesophyll thylakoids. **a** Representative gel patterns of thylakoids (500 µg Chl mL^−1^) solubilised using different detergents and detergent mixtures (given in % w/V), and separated in 4.3–12% gel gradient. Dig: digitonin, *β*-DM: *n*-dodecyl-*β*-d-maltoside, DS: *n*-dodecyl-sucrose. **b** The ratio of the more sensitive bands is given in % of the total PSI, PSII and Lhc amounts, respectively. Complexes are marked as in Fig. 1a. Smear is the bluish green staining at the beginning of the separating gel where bands cannot be noticed clearly given in % of total stain in the lane. Effects of different detergents were compared using one-way ANOVA with Tukey’s multiple comparison test for each group separately [*P* < 0.05; 6 technical repetitions: 3× solubilisation and separation in 2–2 lanes]. Significant differences are indicated by different letters in each group separately

Concerning the mildness of detergents studied, it was found that supercomplexes, particularly PSI-LHCII, were more stable in the presence of digitonin than using *β*-DM or DS (Fig. 3a, b). However, the solubilisation of thylakoids with digitonin only reached about 40–50% and, in addition, more solubilised material applied to the gel remained at the top of the stacking gel and even about 15% appeared as a smear near to the start of the separating gel (Fig. 3b). The band pattern showed the retention of the largest PSII supercomplexes and PSI-LHCII complex, CS complex, and an extra, somewhat larger monomer PSII band containing some polypeptides running a little further to the PsbD,A region was also noticeable (Additional file 1: Fig. S3b). Increasing the concentration of digitonin from 1 to 2% did not cause any difference in the solubilisation efficiency and band pattern in agreement with the fact that digitonin was found to solubilise only the stroma and grana margin lamellae [22].

While the use of digitonin only resulted in a partial solubilisation, *β*-DM was able to solubilise the maize M thylakoids almost completely (> 95%) even at low (0.5%) concentration. However, there was some discrepancy using 0.5% *β*-DM: the amount of Lhc-m was always elevated compared to higher *β*-DM concentrations. The PSI-LHCII supercomplex was very sensitive to *β*-DM. In addition, the elevation of *β*-DM concentration from 0.5 to 2% led to a shift from larger PSII supercomplexes to smaller ones. A mixture of *β*-DM and digitonin gave better results: it also solubilised thylakoids similarly to *β*-DM alone, the large supercomplexes and LHCII-a were retained, PSI-LHCII being the most sensitive but still being retained much better than using only *β*-DM for solubilisation, in agreement with the findings in [10]. LHCII-t was hardly sensitive to the presence of detergents as it was also found earlier [24]. DS proved to be a little harsher than *β*-DM, but also became milder in a mixture with digitonin. It follows, if we can find milder detergents than *β*-DM with good solubilisation ability (an example may be *α*-DM mentioned by Pagliano et al. [61]), and use it together with digitonin, it may be more useful to preserve the native state of complexes.

The best resolution of complexes in thylakoids isolated from mature untreated plants (500 µg Chl mL^−1^, protein/Chl ratio around 4–5) was obtained with 1% *β*-DM and 1% digitonin, and the retention of complexes was not worse than using mixtures of lower *β*-DM concentration (Fig. 3b). Thus, we used this detergent mixture in solubilisation of mature thylakoids. In the case of still developing thylakoids, however, one must take into consideration the high protein content of the samples, too. Lowering the Chl concentration to 250 µg mL^−1^ during solubilisation usually gave satisfactory results if the protein per Chl ratio doubled (e.g. after 16 h greening of etiolated plant material). Checking the protein/Chl ratio is also advised using thylakoids isolated from plants after strong stresses [28].

#### A 4.3–8% BN gel system is beneficial for the separation of the large complexes

**Fig. 4:**
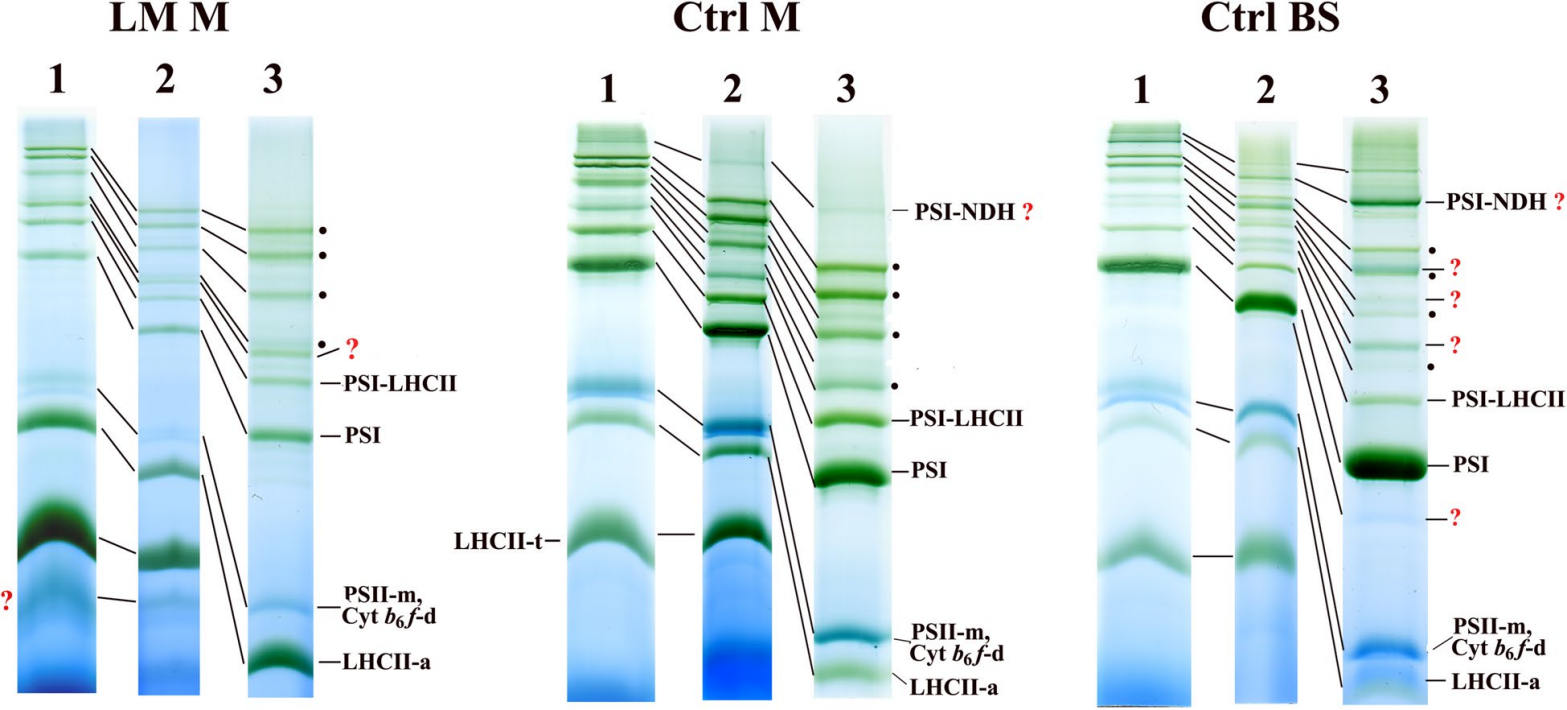
Efficiency of different gel gradients on the BN PAGE separation of mega-/supercomplexes in lincomycin treated maize mesophyll (LM M), untreated maize mesophyll (Ctrl M) and bundle sheath (Ctrl BS) thylakoids. Thylakoids (500 µg Chl mL^−1^) were solubilised using 1% (w/V) *β*-DM plus 1% (w/V) digitonin, and separated in 5–12% (1), 4.3–12% (2) and 4.3–8% (3) gel gradients. Complexes are marked as in Fig. 1a, PSII supercomplexes are marked by points, d: dimer, m: monomer. Complexes identified in the following part of the paper are marked by question marks

Earlier, 5–12% BN gradient gels were used for the separation of thylakoid complexes (e.g. [18, 20, 28]). Later on, higher resolution of megacomplexes was achieved either using gel gradients of 4% starting concentration [34, 37] or so-called large-pore gels ([16], and others working in the same laboratory). Resolution of PSII supercomplexes on CN gels could have been improved by decreasing the end concentration of the separating gel [62]. In our practice, though the resolution of mega-/supercomplexes has already been promoted by the decrease in the starting acrylamide concentration to 4.3%, it was significantly further facilitated by using 4.3–8% gradient gels (Fig. 4). It allowed large complexes to run further into the gel resulting in higher resolution of the bands in the PSII-m and (PSI-NDH-mc) region, while the resolution of bands larger than PSI-NDH-mc was perhaps slightly lower than in large-pore gels [16]. In addition, the presence of glycerol in all parts of the gel contributed to the preservation of the native state of complexes (even in denaturing gels—see Additional file 1: Fig. S1b).

Henceforth, 4.3–12% gels were used aiming at the separation of all thylakoid complexes in 1st and 2nd dimension BN/CN PAGE, but we employed 4.3–8% gels to obtain better separation of complexes in the mega-/supercomplex region.

### Example 1: Characterisation of large PSI complexes

**Fig. 5:**
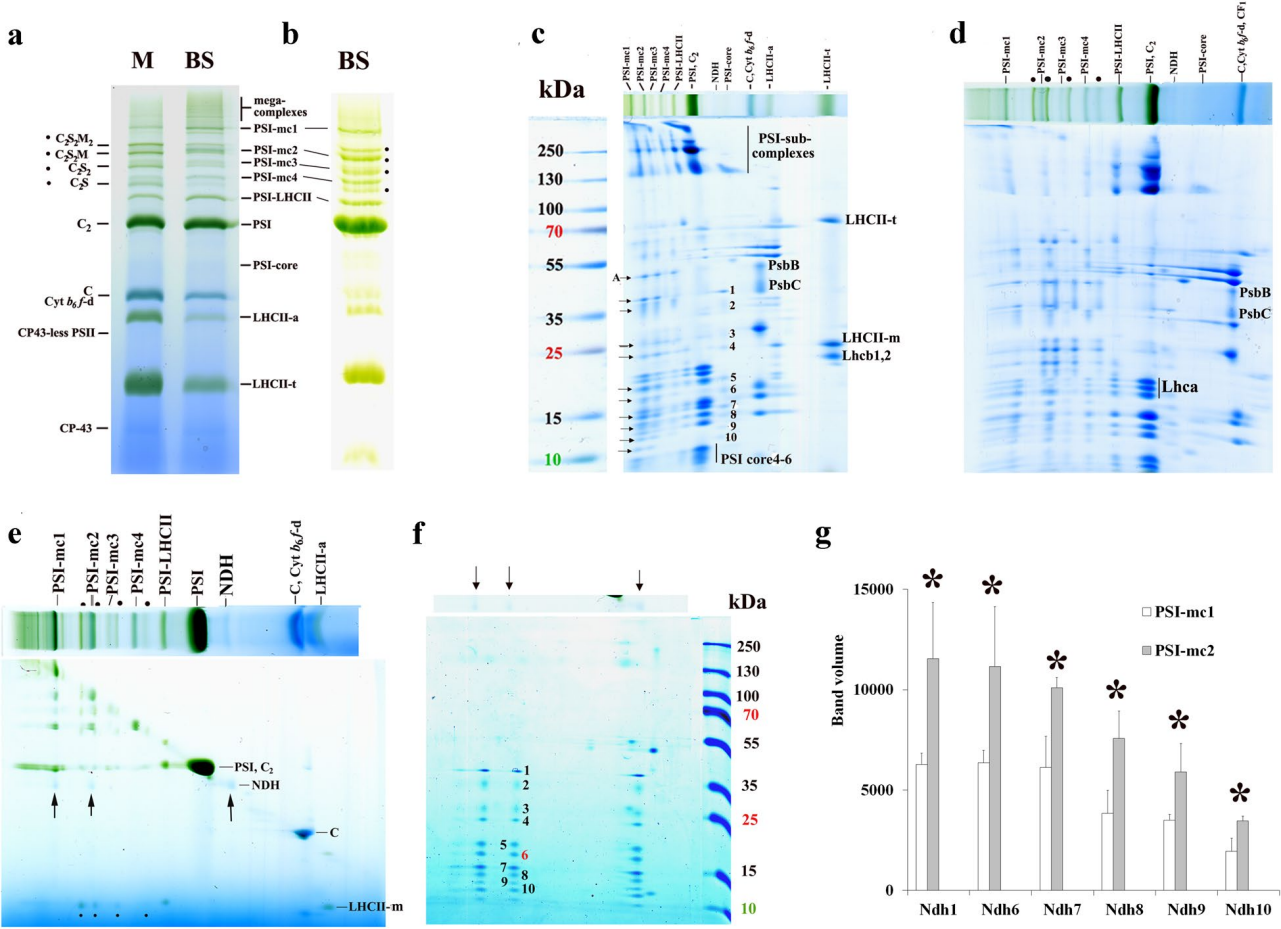
Characterisation of untreated maize mesophyll (M) and bundle sheath (BS) thylakoid complexes. **a** BN PAGE and **b** CN PAGE separation of complexes from M (**a**) and BS (**a**, **b**) thylakoids. **c**, **d** Polypeptide patterns of BS thylakoid preparations of different purity (more or less successful separation from M thylakoids, see the Chl *a/b* ratios given below). PSI megacomplexes (PSI-mc) are numbered, other complexes are marked as in Fig. 1a. PSII supercomplexes are marked with points. The polypeptides of the NDH complex are numbered (**c**), and their presence in PSI megacomplexes is marked by arrows. A: pnsB1 (see Table 1) present in PSI megacomplexes, but not present in the free NDH complex. **e** Two-dimensional BN/BN PAGE of BS thylakoids. PSII supercomplexes are marked with points, and NDH complexes separated in the second dimension BN PAGE by arrows. C_2_ and C are PSII-d and PSII-m. **f** Polypeptide pattern (numbered 1–10) of the NDH complexes isolated in the zone marked by arrows in **e**, and obtained by SDS PAGE. Rubisco L and S subunits are also seen in the right side of the pattern. **g** Amounts of the Ndh polypeptides non-overlapping with PSI ones in PSI megacomplexes normalised to PSI core 4–6 (see in **c**). Differences in each polypeptide between PSI-mc1 and PSI-mc2 were compared using multiple t-tests [*P* < 0.05; n = 3 × 2 (biological × technical)]. Significant differences are marked with stars (*). The Chl *a/b* ratio of the used M and BS (**a**, **b**), BS1 (**c**, **e**), and BS2 (**d**) thylakoids were 3.62, 6.27, 6.97, and 5.95, respectively. Thylakoids (500 µg Chl mL^−1^) were solubilised using 1% (w/V) *β*-DM plus 1% (w/V) digitonin in the first-dimensional BN/CN PAGE, and with 1% (w/V) *β*-DM in the second-dimensional BN PAGE. Complexes were separated in 4.3–12% (**a**–**c**, **g**—first dimension, **e**—second dimension) and 4.3–8% (**d**, **e**—first dimension) BN/CN gel gradients followed by SDS PAGE to obtain the polypeptide patterns of bands (**c**, **d**, **f**, **g**). Standard proteins: PageRuler™ Plus Prestained Protein Ladder (ThermoFisher Scientific 26619, Lot #00803392)

While in maize M thylakoids PSII supercomplexes dominated the BN gel region above the PSI band, mainly PSI-mc bands could be seen in BS thylakoids (Fig. 5). In BS thylakoids, four PSI-mc bands (PSI-mc1–4) were detected running above the PSI-LHCII band together with some faint bands (seemingly also PSI-mc-s). The molecular mass of PSI-mc1–4 complexes calculated from the calibration curve given in Additional file 1: Fig. S4 using internal standards including the molecular masses determined in [9, 63] were about 1740, 1325, 1140, and 935 kDa, respectively. Though PSII-m was the main PSII form in the BS thylakoids, large PSII supercomplexes and PSII-d, supposed to function in redox poise control of cyclic electron transport [64], could be also detected even in the cleanest BS preparations. The band- and polypeptide patterns of the complexes separated by BN or CN PAGE were practically identical (Additional file 1: Fig. S5) as it was also demonstrated by Järvi et al. [16] in *Arabidopsis* plants.

Using higher resolution BN gels [4.3–8% gradient], the PSI mc1–4 bands were separated from PSII-s bands. It was clearly visible in the less clean BS samples of relatively lower Chl *a/b* ratio (Fig. 5d). In *Arabidopsis* thylakoids solubilised with digitonin, BN bands around the position of PSI-mc1 and PSI-mc3 were supposed to be trimeric and dimeric forms of PSI, respectively, and were interpreted as solubilisation artefacts [38]. Later, the PSI-mc1-like band was identified as PSI-NDH-mc [16, 49, 63]. In the more complex first-dimensional BN pattern of maize BS thylakoids, a 525 kDa band appeared migrating below the main PSI band (polypeptide components are marked with numbers in Fig. 5c) which corresponded to the NDH complex of ~ 550 kDa [65–67]. However, subcomplex B of the NDH complex could be hardly detected in the polypeptide pattern of this solubilised complex (Table 1). This complex was shown to accumulate preferentially in BS thylakoids [68, 69]. According to the polypeptide patterns obtained by the second-dimensional BN PAGE followed by SDS PAGE, this complex could be isolated with similar polypeptide pattern and ratios in the free complex, PSI-mc1, and PSI-mc2, and in a larger megacomplex, too (Fig. 5f, Additional file 1: Fig. S6).

**Table 1:** Polypeptides detected in the PSI-mc-s and the NDH complex by MS analysis

	Plant	Protein	M.W	PSI-mc1	PSI-mc2	PSI-mc3	PSI-mc4	NDH
			Da	Score	Score	Score	Score	Score
PSI								
A0A3B6UD72	*Z. m*	psaA	83,055	4357^x,a^	4362^x^	1862^a,x^	1568^x^	
A0A5P8KLV9	*Z. m*	psaB	82,517	5909^x,a^	4607^x^	3917^a,x^	1866^x^	
A0A5P8KLH3	*Z. m*	psaC	8893	15414^x,a^	5503^x^	6691^a^	6269^x^	
A0A317YJL6	*Z. m*	psaD	21,563	41944^x,y^		30676^b,a,x,y^		
B4FAW3	*Z. m*	psaD	21,563		57637^y,x^		18722^x^	
A0A3L6E4J8	*Z. m*	psaE	14,427	32357^x,y^	28888^y,x^	27,812 ^b,a,x,y^	4825^x,a,b^	
A0A096RAV0	*Z. m*	psaF	24,487	30393^y,a^	42839^y,x^	30,393 ^b,a,x,y^	6960^x,y^	
B6U534	*Z. m*	PsaG	15,304	6320^x,a^	5675^x^	5733^a,x^	1368^x^	
A0A3L6E9J9	*Z. m*	psaH	14,920	11956^x^	8266^x^	20186^b,x,y^	756^a^	
A0A3L6E041	*Z. m*	psaK	13,595	3790^x^	3444^x^	3790^x,a^	3697^x^	
A0A3L6DL54	*Z. m*	psaL	22,240	985^x^	829^x^	1183^a^	2609^x^	
A0A3L6FDJ3	*Z. m*	psaN	23,369	3461^x^		2576^a^		
A0A3L6F840	*Z. m*	lhca-P4	29,477	11070^x,a,b^	9737^x^	10327^a,x,y^		
A0A3L6EJZ9	*Z. m*	lhca5	33,854	376^a^				
A0A0F7H1K9	*H. b*	lhca6	29,515		1057^y^			
A0A6M3WH11	*C.p*	lhca6	28,442		1018^y^			
NDH chloroplastic								
e-donor binding								
K7TFI4	*Z. m*	ndhU	25,323	194^a^	7106^x^			3688^x^
A0A3L6GCK8	*Z. m*	ndhU	25,309	7627^x^				
Subcomplex A								
A0A5P8KLE9	*Z. m*	ndhH	45,629	12097^x,a^	12285^x^	479^a^		7474^x^
A0A5P8KLE6	*Z. m*	ndhI	21,143	27850^x,a^	13488^x^			15901^x^
A0A5P8KLA0	*Z. m*	ndhJ	18,657	7105^x,a^	26589^x^			18907^x^
A0A5P8KLC5	*Z. m*	ndhK	27,902	11795^x^	7443^x^			5595^x^
B4FRX4	*Z. m*	ndhM	23,964	17739^x^	11325^x^			6239^x^
A0A3L6FA99	*Z. m*	ndhN	23,024	2640^x,a^	17139^x^			9570^x^
A0A3L6DTN1	*Z. m*	ndhO	16,595	12097^x,a^	3167^x^			3296^x^
Subcomplex B								
A0A3L6GBC8	*Z. m*	pnsB1	51,403	15044^y,x,a,b^	13418^x,y^	4167^a,b,x,y^	4529^x,a,b,y^	
A0A3L6DA24	*Z. m*	pnsB2	37,902	32626^x,a,b,y^	23464^y^	8554^a,b,x,y^	2260^b,a^	
B4FWG2	*Z. m*	pnsB2	37,902		27745^x^		15228^x^	
A0A3L6E2Y7	*Z. m*	pnsB3	20,798	4649^x,y^	5414^x^			
A0A1D6HK95	*Z. m*	pnsB4	31,198			1985^x^		
A0A1D6HK96	*Z. m*	ndhB4	20,603	2637^y^		2637^b^		
A0A3L6FDN2	*Z. m*	pnsB5	25,605	10998^y,x^	10306^x^	1864^a,x,y^	7131^x,a,b^	
Membrane subcomplex								
A0A3B6UD78	*Z. m*	ndhA	40,346	579^a^		909^a^		
P25706	*Z. m*	ndhA	40,485	5094^x^	6331^x^			5854^x^
A0A5P8KL97	*Z. m*	ndhC	13,851	1596^x^	1606^x^			
A0A5P8KM09	*Z. m*	ndhD	56,316	3444^x,a^	4815^x^	503^x,a^	985^x^	
A0A5P8KLF2	*Z. m*	ndhF	82,950	5788^x,a^	6092^x^	2561^a,x^	2077^x,a^	
Lumenal subcomplex								
A0A1D6JYG6	*Z. m*	pnsL1	25,683	10829^y,a,b,x^	10571^x,y^	5036^b,a,x^		6759^x,y^
A0A1D6HBD7	*Z. m*	pnsL2	24,200	27466^y,a,b,x^	20585^x,y^	4104^b,a,x,y^		15443^x,y^
A0A3L6E7E6	*Z. m*	pnsL3	25,686	5953^y^	5382^x^	2365^b,a^	1008^a^	
A0A1D6HQR2	*Z. m*	pnsL3	28,290		5957^y^		879^b^	

Data were obtained from bands originating from two different isolations and separations. Analysis was performed in maize (mark *a*) and PSI-NDH (mark *b*) databases (isolation 1, separation in 4.3–12% BN gel gradient) and maize (mark *x*) and PSI-NDH (mark *y*) databases (isolation 2, separation in 4.3–8% BN gel gradient) acquired form UniProtKB. Search results can be found in Additional file 3: MS results 1 (isolation 1) and Additional file 4: MS results 2 (isolation 2). Superscripts show the hits. The highest scores (first superscript marks) are shown. Nomenclature of Shikanai [72] was used in the case of chloroplast NDH. The pnsB1 present in PSI-mc-s but not in the free NDH complex and ndhH corresponds to band A and band 1, respectively, in Fig. 5c

CA: carbonic anhydrase; *C.p*.: *Carica papaya*; FS: iron-sulphur protein; FV: flavoprotein; *H.b*.: *Hypseocharis bilobata*; su: subunit; *Z.m*.: *Zea mays*

Comparing the amounts of polypeptides in the different PSI complexes normalised to PSI core bands, the band volumes and ratios of PSI polypeptides were similar in PSI, PSI-LHCII and PSI-mc1,2,4 (Additional file 1: Fig. S7). However, using the same normalisation method elevated amount of Ndh polypeptides were found in PSI-mc2 compared to PSI-mc1 (Fig. 5g). According to the molecular mass of PSI (~ 580 kDa) and NDH (~ 550 kDa), the PSI-mc1 and PSI-mc2 may represent PSI/NDH ratio of two and one, respectively. PSI megacomplexes with different PSI/NDH ratio of two and more were also detected earlier [34, 37, 43, 48, 49, 70, 71]. In agreement with above mentioned data, the relationship of PSI-mc1,2,4 bands obtained by the second-dimensional BN PAGE verified that the solubilisation products of PSI-mc1 were PSI-mc1,2,4, and NDH complex and those of PSI-mc2 PSI-mc2,4 and NDH complex (Fig. 5e). To the contrary, no NDH spot was observed in the pattern of PSI-mc4. A smaller NDH related spot (NDH part) originating from this megacomplex can be suspected on the stained second-dimensional BN pattern of this complex (Additional file 1: Fig. S8). The polypeptide composition of the PSI-mc1,2,4 determined by mass spectrometry supported their PSI-NDH complex characteristics (Table 1). While PSI-mc1,2 contained all the main components of the NDH complex, the polypeptides of subcomplex A apparently were not present in PSI-mc4. PSI-mc2 also contained PSII polypeptides due to its close running to C_2_S_2_M (see Additional files 3 and 4).

**Fig. 6:**
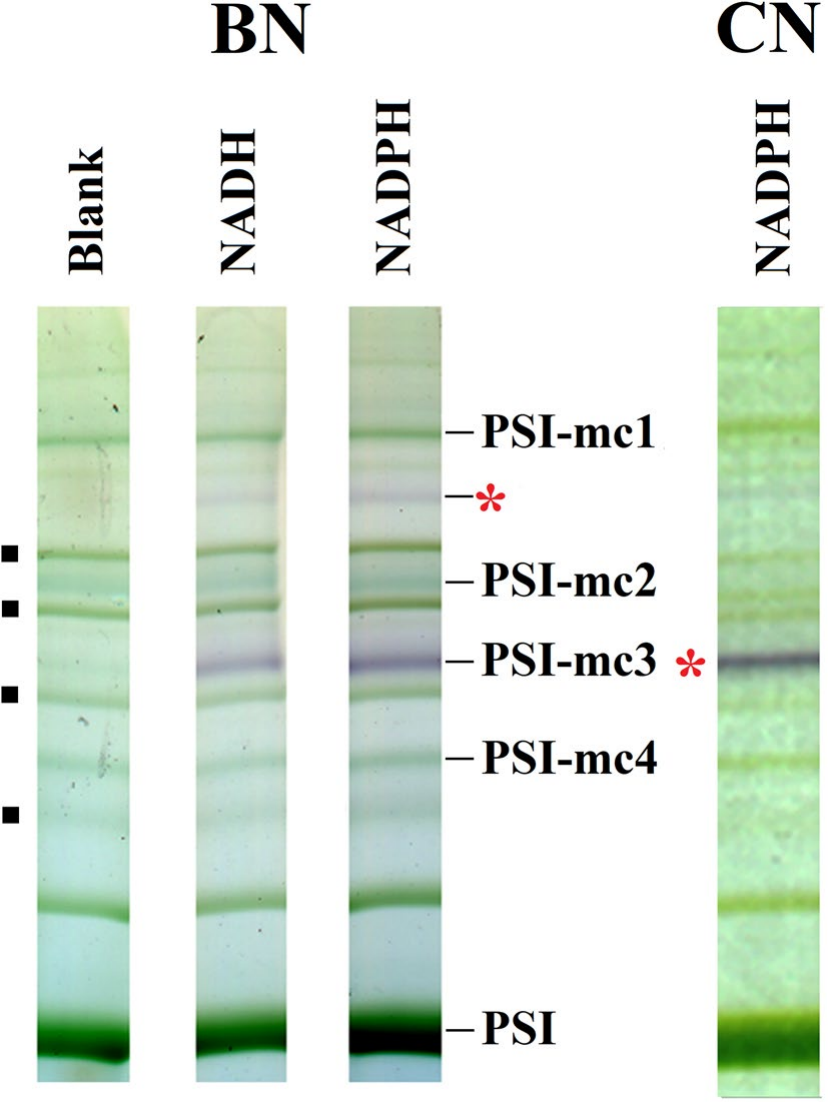
NADH and NADPH dehydrogenase activity of the maize BS megacomplexes detected in BN and CN gels. *: Bands showing dehydrogenase activity. Points refer to PSII supercomplexes. Thylakoids (500 µg Chl mL^−1^) were solubilised using 1% (w/V) *β*-DM plus 1% (w/V) digitonin and separated in 4.3–8% BN/CN gel gradients

The polypeptide composition of the PSI-mc3 band, appearing in low amount, was not as clear as that of other PSI megacomplexes. Nevertheless, many polypeptide components of PSI and chloroplast NDH were present (Table 1). PSI-mc3 also contained PSII polypeptides due to its close running to C_2_S_2_. In chloroplasts, PSI-NDH megacomplexes are known to utilise ferredoxin as reductant [72–74] bound to the complex by the NdhS protein that shares structural similarity with PsaE [75]. As subcomplex A was lacking and electron donor binding polypeptides were not detected in PSI-mc3 (Table 1), and enzymes of similar size with NAD(P)H dehydrogenase activity were discovered in chloroplasts of rice (*Oryza sativa*) flag leaves and panicles [69], we investigated NAD(P)H dehydrogenase activity of megacomplexes. Among the main PSI megacomplexes, only PSI-mc3 band showed high dehydrogenase activity (Fig. 6). The detected activity was similar with either NADH or NADPH. Faint activity could be also seen in a band of larger molecular mass running between PSI-mc1 and C_2_S_2_M_2_ complex. Activity-stained complexes were the same in BN and CN gels. NAD(P)H dehydrogenase activity of the PSI-mc3 band may come from contaminating mitochondrial complex I (~ 1000 kDa) [76] since many polypeptide components of mitochondrial NADH dehydrogenase [77] were also detected (Table 2). However, NAD(P)H-dependent NDH complexes of similar size were detected in rice [78], and a dimeric NDH related complex of ~ 1000 kDa was also found in BS chloroplasts functioning in the carbon concentration mechanism [67]. PSI dimers of similar size were also detected in BN PAGE [38] and by single particle analysis [43]. Interestingly, Proton Gradient Regulation 5 (PGR5) was also detected in PSI-mc3 (see Additional file 3). Different complexes of similar molecular mass present in the same band cannot be excluded in BN PAGE separations. The detailed composition of this band needs further examination.

**Table 2:** Mitochondrial complex I components found in PSI-mc3

Accession	Plant	Protein	M.W	PSI-mc3
			Da	score
Mitochondrial complex I				
A0A1D6L210	*Z.m*	FS1	80,666	22069^a,x^
B4FGH7	*Z.m*	FS4	17,250	1834^a^
A0A1D6NDN3	*Z.m*	FS7	13,933	3128^a,x^
A0A3L6G2J2	*Z.m*	FV1	55,160	6878^a^
B6T6U3	*Z.m*	FV1	55,146	1864^x^
B4FPX5	*Z.m*	FV2	35,177	1867^a^
Q6R9J9	*Z.m*	nad2	53,634	1035^a^
Q6R9H4	*Z.m*	nad5	74,440	2815^a^
Q5K097	*Z.m*	nad9	22,916	12347^a,x^
K7USN8	*Z.m*	CA1	28,401	549^a^
A0A3L6DZ16	*Z.m*	CA2	29,625	3062^a,x^
A0A3L6DF13	*Z.m*	alpha5	24,897	1701^a^
A0A317YH98	*Z.m*	alpha6	15,131	396^a^
A0A1D6HPC4	*Z.m*	alpha9	37,931	6035^a,x^
B4FCV1	*Z.m*	alpha12	18,523	3914^a^
A0A317YFY5	*Z.m*	alpha13	15,963	5430^a^
A0A317Y753	*Z.m*	beta10	12,493	2733^a^
B6TLX0	*Z.m*	10.5 kDa su	11,094	4786^a^
A0A1D6GG96	*Z.m*	23 kDa su	25,650	8236^a^
B6TMG9	*Z.m*	29 kDa su	27,872	1448^a^

Separations, MS analyses, and presentation were done as in Table 1

**Fig. 7:**
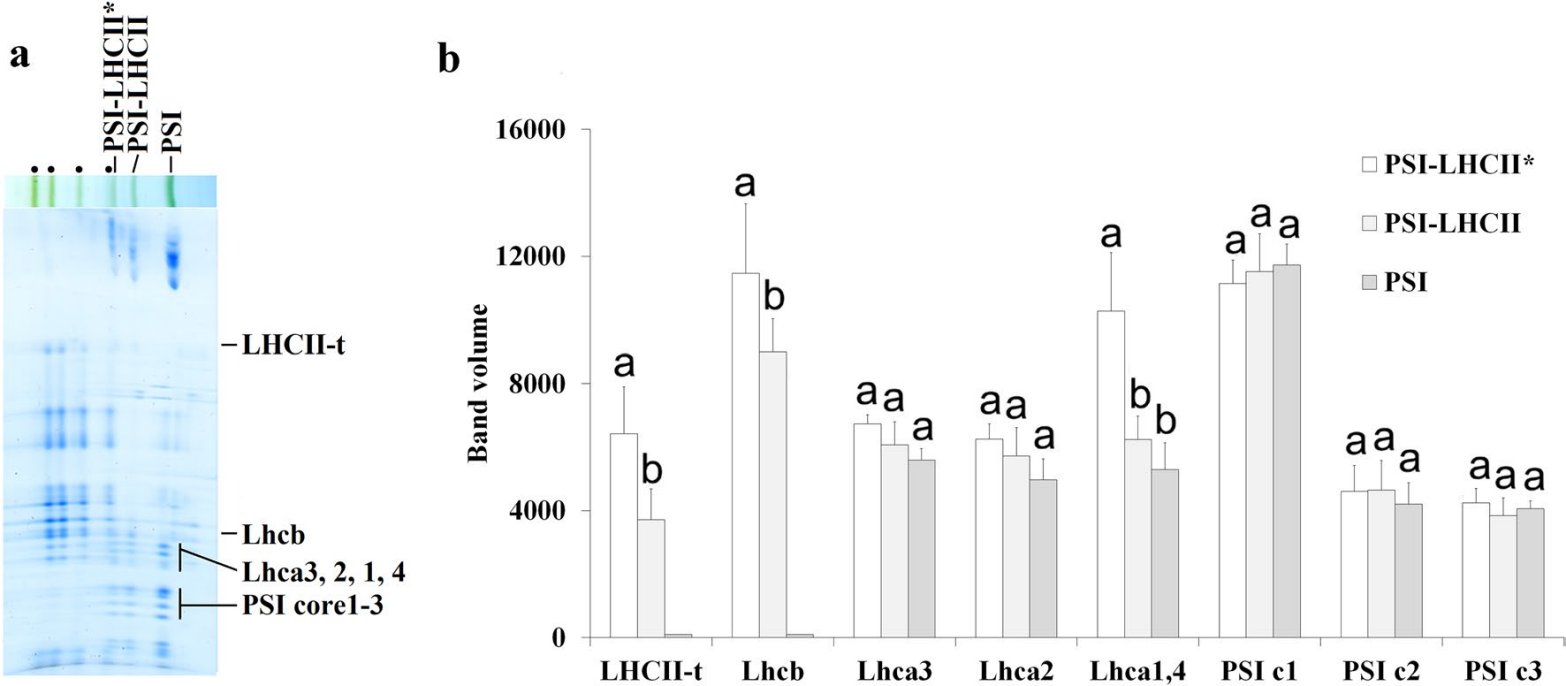
Polypeptide patterns of PSI-LHCII supercomplexes separated from mesophyll thylakoids of lincomycin treated maize. **a** BN/SDS gel pattern of thylakoids. Points refer to PSII supercomplexes. Thylakoids (500 µg Chl mL^−1^) were solubilised using 1% (w/V) *β*-DM plus 1% (w/V) digitonin, and complexes were separated in 4.3–8% gel gradients followed by SDS PAGE. PSI-LHCII*: large PSI-LHCII band. **b** Comparison of the polypeptide profiles of PSI and PSI-LHCII supercomplexes normalised to PSI core 1–3 (see in **a**. Variations in the amounts of polypeptides in the different complexes were compared using two-way ANOVA with Tukey’s multiple comparison test [*P* < 0.05; n = 2 × 3 (biological × technical)]. Significant differences are indicated separately in each polypeptide/LHCII-t group by different letters

In maize M thylakoids isolated from LM treated plants another large PSI complex (~ 825 kDa) was discovered by two-dimensional BN/SDS PAGE (indicated by asterisk in Fig. 7a) which was sometimes also observed in the untreated control ones (*cf*. Additional file 1: Fig. S9a). The polypeptide composition of the new PSI-s differed from that of the PSI-mc bands detected in BS thylakoids, the former being more like to that of PSI-LHCII. The PSI-LHCII band contains one copy of all Lhca1–4 complexes and an LHCII trimer [39]. There are literature data on larger PSI complexes binding more LHCII trimers [23, 42–45]. However, small differences in the LHCII-t and Lhcb content between PSI-LHCII and PSI-LHCII* may be rather due to the partial overlap of the latter with the C_2_S complex. Otherwise, PSI-LHCII* showed totally similar composition and position in the separation pattern to the PSI-core-6Lhca-LHCII complex isolated from *Arabidopsis*, which binds an extra Lhca1,4 dimer, and was found more abundant in State 2 [79]. Indeed, the Lhca1,4 content was also higher in PSI-LHCII* complex (Fig. 7b).

Summing up the results about the composition of large-mass PSI bands in maize thylakoids, complexes proved to be differently solubilised PSI-NDH megacomplexes in which PSI binds either total NDH (PSI-mc1,2) or a part of it (PSI-mc4) or PSI complexes with different antenna complements (PSI-LHCII, PSI-LHCII*). To clarify the nature of the PSI-mc3 band needs better resolution of this gel region.

### Example 2: Quantitative comparison of complexes in different thylakoid samples

From the quantitative point of view, we may compare the changes in the amounts and interactions of thylakoid complexes in the different samples. Before comparison, the sum band volume of all BN lanes must be normalised to the same value because they may differ due to subtle differences in solubilisation or pipetting, see also in Fagioni et al. [27]. If the analysis is free of these problems, then lanes of the same Chl content isolated from mature plants, having a protein/Chl ratio of 4.19 ± 0.46, gave very similar summa volumes [28].

Depending on our interest/purpose, the changes in the amounts of complexes can be demonstrated in multiple ways. One possibility is to display their absolute amount, i.e. how the Chl content present in g fresh or dry weight, per cm^2^ leaf area, in the whole leaf or plant is distributed among the complexes. It means that the summa band volume of the lane corresponds to the Chl concentration or Chl content of the leaf or plant which is divided among the complexes according to their ratios. It seems to be more suitable to use CN PAGE for this purpose because of the different Chl per protein ratios of complex bands. Interestingly, however, there were no large differences between the band volumes of complexes obtained by CN and BN PAGE (Additional file 1: Fig. S10) except in the case of PSII-m, the amount of which at least doubled in the BN PAGE pattern due to its low Chl/protein content and the presence of other complexes (Cyt *b*_6_*f*-d, CF_1_). However, this presentation is influenced not only by changes in the distribution of complexes but also by other parameters such as the fresh or dry weight or area differences or growth characteristics of the leaf or plant.

To get a clear picture on alteration of the composition or complex interactions in thylakoids isolated from multiple or differently treated plants, it is better to compare either the amount of similar complexes or their percentage of a given sample (e.g. control) in samples normalised to the same total volume per lane. This latter plotting enables a better distinction of variations in the thylakoid composition. The ratio of complexes and the distribution among the different forms of a complex may be also important. However, it must be kept in mind that the solubilisation conditions of thylakoid being in different developmental/acclimation stage may not be the same, and varying the detergent concentration also causes changes in the complex interactions.

**Fig. 8:**
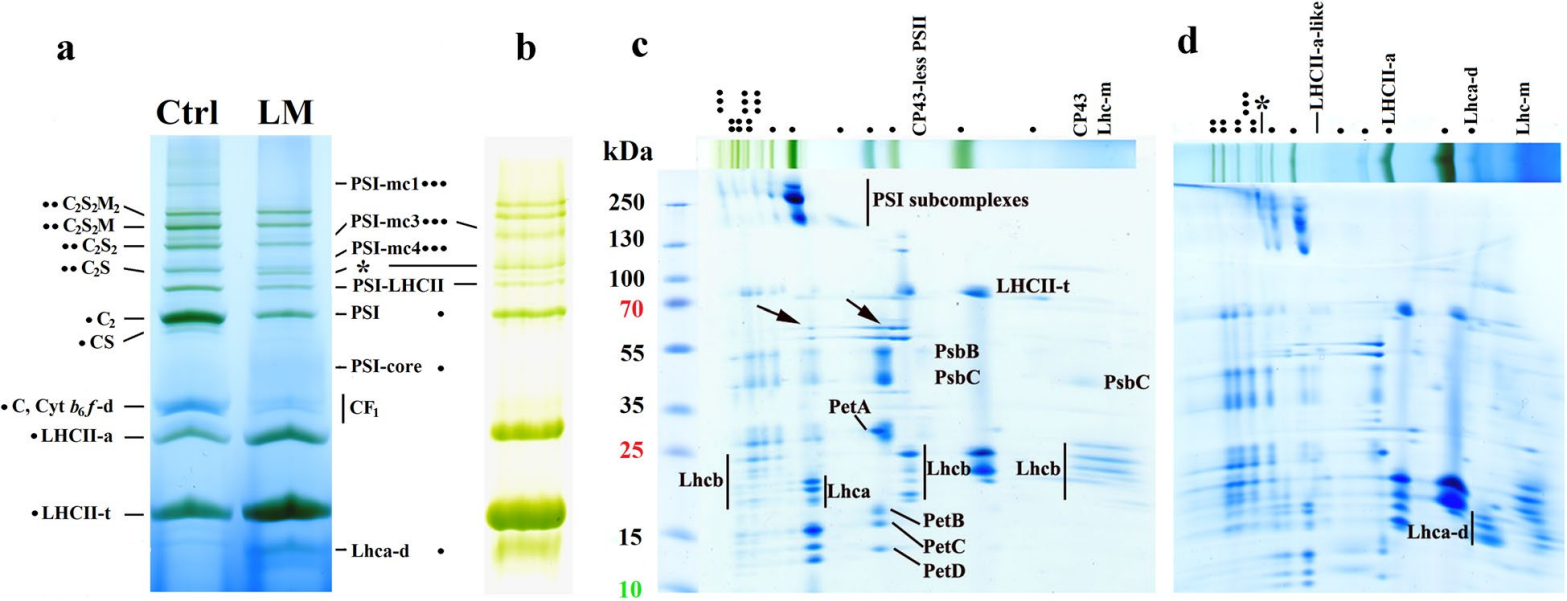
Characterisation of thylakoid complexes in untreated (Ctrl) and lincomycin treated (LM) maize leaves. Complexes obtained by BN PAGE (**a**) and CN PAGE (**b**) separations. Complexes are marked as in Fig. 1a, mc: megacomplexes, *: PSI-LHCII^*^. Polypeptide patterns of untreated (**c**) and LM treated (**d**) thylakoid complexes. Standard proteins: PageRuler™ Plus Prestained Protein Ladder (ThermoFisher Scientific 26619, Lot #00803392). Thylakoids (500 µg Chl mL^−1^) were solubilised using 1% (w/V) *β*-DM plus 1% (w/V) digitonin, and complexes were separated in 4.3–12% gel gradients followed by SDS PAGE

**Fig. 9:**
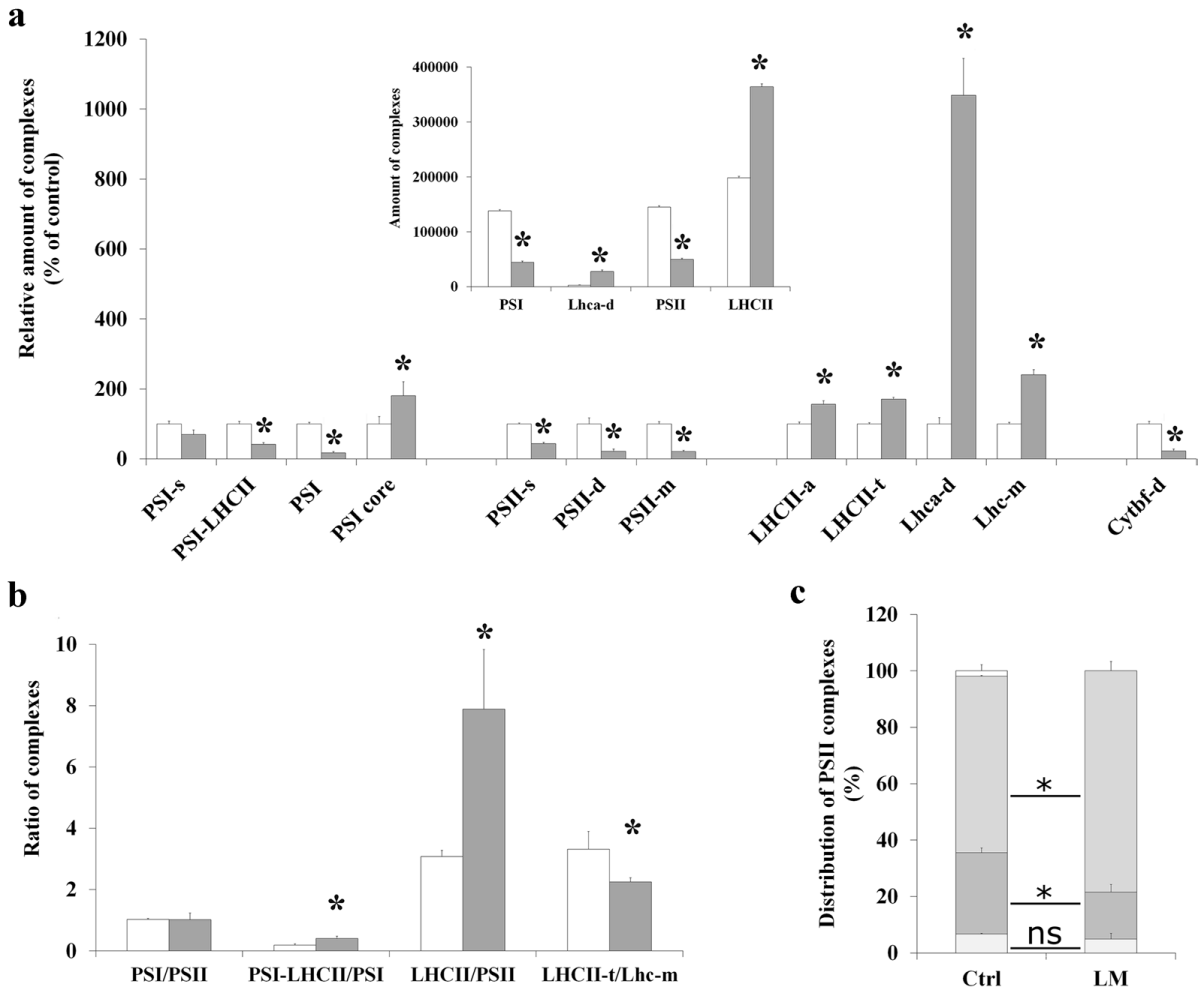
Amounts, ratios, and distribution of complexes in untreated and lincomycin treated maize mesophyll thylakoids. Complexes are marked as in Fig. 1a, s: supercomplex, d: dimer, m: monomer. Thylakoids were solubilised (500 µg Chl mL^−1^) using 1% (w/V) *β*-DM plus 1% (w/V) digitonin and separated in 4.3–12% BN gel gradient followed by SDS PAGE. **a** Relative amount of complexes in thylakoids isolated from untreated (white) and lincomycin treated leaves (grey) given as the percentage of controls. Differences were compared using two-way ANOVA with Bonferroni’s multiple comparison test [*P* < 0.05; significance is indicated by stars (*)]. Inset: Distribution of total band volumes among the complexes. Differences were compared using multiple t-tests [*P* < 0.05; significance is indicated by stars (*)]. **b** Ratio of complexes. The PSI/PSII, PSI-LHCI/PSI, and Lhc-m/LHCII-t ratios were calculated from the BN bands, i.e. PSI and PSII contained the bound antennae, while LHCII/PSII was calculated from the SDS gel pattern: all Lhcb spots and PsbB spots were taken into account (see Additional file 2). Differences were compared using multiple t-tests [*P* < 0.05; significance is indicated by stars (*)]. **c** Distribution of PSII complexes (PsbB in SDS PAGE pattern to eliminate the effects of different antenna sizes): PSII-d (light grey), PSII-m (dark grey), PSII-s (medium grey). In controls some CP43-less PSII was also present (white on the top). Differences were compared using multiple t-tests [*P* < 0.05; significance is indicated by stars (*)]. In all cases, n = 2 × 3 (biological × technical)

As an example, the amount of the complexes and the complex interactions of M thylakoids of LM treated maize plants were compared to those of controls. The Chl content of treated leaves decreased only moderately to about 73% of the control (from 1648 ± 140 to 1205 ± 235 µg Chl g^−1^ FW). However, the Chl *a/b* ratio was strongly lowered: from 4.03 ± 0.04 and 3.51 ± 0.12 in the controls to 2.90 ± 0.22 and 2.56 ± 0.22 in LM treated leaves and M thylakoids, respectively. In agreement with these data, granum structures (Additional file 1: Fig. S11, see also in [80, 81]) and light-harvesting complexes were much more abundant in M chloroplasts of LM treated, than in untreated control plants (Fig. 8a, b), and even free Lhca dimers were present (Fig. 8a, b, d, see also Additional file 1: Fig. S12). Interestingly, a complex of similar polypeptide pattern to LHCII-a running a little further than CS (probably LHCII-a–d) was also observed in LM treated thylakoids, which may originate from PSII megacomplexes in which M-trimers are interacting [62]. The ratio of complexes active in the electron transport (PSI, PSII, Cyt *b*_*6*_*f*) significantly decreased, while that of the antennae increased compared to control (Fig. 9a) leading to an unchanged PSI to PSII, and elevated antennae per PS-core ratios (Fig. 9b). The shift of PSII forms to PSII supercomplexes and the higher PSI-LHCII/PSI and Lhc-m/LHCII-t ratio (Fig. 9b, c) may be related to the maintenance some photosynthetic activity and/or operation of different protective mechanisms in M thylakoids of LM treated plants [33] which may sustain the viability of plant.

## Conclusions

Solubilisation of thylakoids with 1% (w/V) *β*-DM plus 1% (w/V) digitonin and increasing the resolution of the large complexes using 4.3–8% BN gel gradient proved to be a powerful method for the separation of thylakoid mega- and supercomplexes in their native state as evidenced by the characterisation of large PSI complexes in control BS and LM treated M maize thylakoids. For quantification of complexes, appropriate sample application, adequate baseline correction, and deconvolution of complexes running in the same band are necessary. To compare the amounts of thylakoid complexes correctly in varying samples, ways of comparison the absolute/relative amounts of complexes and distribution among their different forms are proposed.

## Methods

### Plant materials

Plants used for experiments were as follows. Wheat (*Triticum aestivum* L. cv. Mv Béres) and maize (*Zea mays* L. cv. Mv350) were grown in hydroponics (¼ strength Hoagland solution changed every 2 days) for 11 and 14 days, respectively, in a climate chamber (14/10 light/dark period, photosynthetic photon flux density (PPFD) of 200–250 μmol photons m^−2^ s^−1^, 23 °C and ~ 75% relative humidity). Oilseed rape (*Brassica napus* L. cv. DK Exquisite) was grown under similar conditions in a modified half-strength Hoagland solution [82]. Poplar plants (*Populus jacquemontiana* Kimura var. *glauca* Haines cv. Kopeczkii) were grown hydroponically as it is given in Solti et al. [83]. For LM treatment, maize (*Zea mays* L. cv. Mv NK 333 TC) seedlings were grown in darkness for 5 days and then greened for 72 h with continuous illumination of 100 μmol photons m^−2^ s^−1^ PPFD. LM (Sigma, 100 µg mL^−1^) was added into the nutrient solution 16 h before the start of illumination and was present during the whole greening process. Other plants were cultivated in the proper soil cultures: *Haberlea rhodopensis* Friv., shade ecotype at 25 μmol photons m^−2^ s^−1^, *Primula auricula* L. at ambient illumination in greenhouse, and *Jatropha curcas* L. in the same climate chamber and conditions as mentioned above.

### Isolation of thylakoids

Poplar and *H. rhodopensis* thylakoids were isolated as it is written in Sárvári and Nyitrai [7] and in Georgieva et al. [84], respectively. Leaves of other plants were homogenised in 50 mM 4-(2-hydroxyethyl)-1-piperazineethanesulphonic acid (HEPES)-KOH, pH 7.0, 330 mM sorbitol, 2 mM ethylenediaminetetraacetic acid (EDTA), 2 mM MgCl_2_, 0.1% (w/V) bovine serum albumin (BSA), 0.1% (w/V) Na-ascorbate at 4 °C for 2 × 3 s by Waring blender. The homogenate was filtered through four layers of gauze and two layers of Miracloth™ (Calbiochem-Novabiochem, San Diego, CA, USA). Chloroplasts were immediately pelleted by centrifugation (1500×g, 5 min, 4 °C). The chloroplast pellet was resuspended in washing buffer (50 mM HEPES–KOH pH 7.0, 330 mM sorbitol, 2 mM MgCl_2_). Osmotic shock and removal of CF_1_ according to Fuad et al. [85] was carried out as described previously [7]. For isolation of BS thylakoids from maize leaves, the material remaining on the filter after a short homogenisation to isolate M plastids was homogenised two times in fresh medium for 30–30 s, then filtered and rinsed, and at last the BS strands remaining on the filter were homogenised further in a mortar, then thylakoid isolation followed as it was mentioned above. Thylakoid samples were stored in 2 mM tris(hydroxymethyl)aminomethane (Tris)-maleate pH 7.0, 35% (w/V) glycerol in liquid nitrogen. They can be stored for years without change under these conditions, but they must be stored in aliquots used once for solubilisation and native PAGE.

### Determination of chlorophyll content

Pigments were extracted from leaf disks or thylakoids with buffered (5 mM *N*-(tri(hydroxymethyl)methyl)glycine (Tricine)-KOH pH 7.8) 80% (V/V) acetone at low light. The extracts were centrifuged at 4 °C with 10,000×*g* for 5 min and measured spectrophotometrically by a UV–VIS spectrophotometer (UV-1601, Shimadzu, Japan). Chl content was calculated according to Porra et al. [86].

*PAGE methods* (see also Additional file 2: BN-solubilisation and evaluation; Additional file 5: Gel preparations and buffers).

To separate thylakoid complexes, gel electrophoresis was performed under native conditions by BN-PAGE [12] based on the method of Schagger and von Jagow [11] but using Mini-Protean apparatus (BioRad) and 4% (w/V) stacking and 5–12% or 4.3–12% or 4.3–8% (w/V) acrylamide gradient separating gels of 1.5 mm, all containing 8.7% (w/V) glycerol. After washing by 10 min centrifugation with 10,000×*g* in washing buffer (50 mM BisTris-HCl pH 7.0, 330 mM sorbitol, 250 µg mL^−1^ Pefabloc), thylakoids (500 µg Chl mL^−1^) were solubilised in solubilising buffer (750 mM aminocaproic acid [ACA], 50 mM Bis–Tris, pH 7.0, 0.5 mM EDTA) with 1% (w/V) *β*-DM (SIGMA) plus 1% (w/V) digitonin (SERVA) or other detergents/mixtures including DS (Calbiochem). Two-fold concentrated stock (2 × SB) supplemented by a corresponding volume of deionised water to the volume of the thylakoid samples was applied in order to achieve the final concentration of the solubilisation buffer and Chls. After resuspending the thylakoid pellet with pipette, solubilisation was performed after adding the chosen detergent (see also Additional file 2, sheet “solubilisation”) on ice for 30 min with three short vortexing meantime. After 15 min centrifugation with 18,000×*g* at 4 °C, the supernatant was supplemented with 1/5 volume of glycerol for CN PAGE or 5% (w/V) Serva Blue G (SBG) dissolved in 500 mM ACA for BN PAGE, and 10–20 µL of the solubilised material was applied per lanes. To remove residual ammonium persulphate from the wells, sample places were rinsed with cathode buffer for CN PAGE and cathode buffer containing 5-times diluted SBG (that enables a more convenient sample application) for BN PAGE before sample loading. CN PAGE was performed as BN PAGE except that cathode buffer contained 0.02% (w/V) *β*-DM and 0.05% (w/V) sodium deoxycholate (DOC) instead of SBG.

For the second-dimensional BN PAGE mainly according to Rantala et al. [17], gel strips were solubilised using 1% (w/V) *β*-DM in solubilising buffer for 30 min in ice by shaking, then rinsed and placed to the top of a gradient gel (4.3–12%) in solubilising buffer containing 1% (w/V) *β*-DM, 5% (w/V) SBG, and 0.5% (w/V) agarose. Agarose was dissolved by heating. Warm agarose cooled down immediately during pipetting around the gel slice. Cathode buffer contained 0.02% (w/V) SBG plus 0.02% (w/V) *β*-DM.

Electrophoresis was carried out at 6 °C with a maximum of 5 mA per gel (1 cm^2^ gel surface) with constant voltage of 50 V (30 min), 100 V (30 min), 150 V (30 min), and after changing the upper buffer to one without SBG or blue cathode buffer with 1000× diluted SBG (it was more convenient) in BN PAGE and using the original cathode buffer in CN PAGE, with 200 V (about 2 h).

NAD(P)H dehydrogenase in gel activity was detected as in Quiles et al. [87].

The polypeptide pattern of thylakoids and thylakoid complexes was determined by SDS-PAGE according to Laemmli [52] but modified by using 5% stacking and 10–18% linear gradient separating gels, all containing 8.7% (w/V) glycerol. To obtain the polypeptide patterns of thylakoid complexes, gel strips of about 3 mm wide were cut out of BN-PAGE lanes and attached to the top of the denaturing gel in solubilising buffer containing 0.5% (w/V) agarose. Proteins were separated in the above-mentioned apparatus with a constant current of 20 mA per gel for about 2 h. Following the electrophoresis, gels were stained with the Blue-Silver method [88]. Some polypeptide spots determining the identity of the complexes unequivocally could be recognised on the basis of previous MS determinations of bands run under similar conditions as it was done in Basa et al. [28].

The gels were scanned using an Epson Perfection V750 PRO scanner. Densitometry analysis of gels (i.e. integration of the density of pixels as band volumes on grayscale, not adjusted images) was carried out using the Phoretix image analysis software (Phoretix International, Newcastle-upon-Tyne, UK).

### Determination of protein content

Protein standards (bovine serum albumin, 66 kDa; ovalbumin, 45 kDa; glyceraldehyde-3-phosphate dehydrogenase, 36 kDa; carbonic anhydrase, 29 kDa; trypsinogen, 24 kDa; trypsin inhibitor, 20 kDa; α-lactalbumin, 14 kDa) containing 5 µg protein were run together with solubilised (30 min at room temperature) thylakoids in SDS gels, and their total lane volumes were compared.

### Western blot

Membrane proteins separated by SDS-PAGE were transferred to HyboundTM-C Extra (Amersham-Pharmacia, Piscataway, NY, USA) nitrocellulose membranes in 25 mM Tris pH 8.3, 192 mM glycine, 20% (V/V) methanol and 0.02% (w/V) SDS at 4 °C using 90 V constant voltage (< 0.4 A) for 3 h. Membranes were decorated with rabbit polyclonal antibodies against Lhcb-s (a gift from Dr. Udo Johanningmeier, Bohum Universität, Germany) or with Agrisera AG (Vännäs, Sweden) antibodies against Lhca1 (AS01 005), Lhca2 (AS01 006), Lhca3 (AS01 007) and Lhca4 (AS01 008]). Antibody detection was carried out as in Solti et al. [89].

### Proteomics

#### Sample preparation

Gel band or spots cut out from BN or SDS gels were destained in 25 mM ammonium bicarbonate (AMBIC)/50% acetonitrile (ACN), reduction was performed in 10 mM tris-(2-carboxyethyl)-phosphine at 56 °C for 30 min, then alkylation with 55 mM iodoacetamide at room temperature for 30 min in dark. Gel pieces were washed using 25 mM AMBIC/50% ACN and dried using SpeedVac. Gel pieces were then rehydrated using 5 ng µL^−1^ trypsin in 25 mM AMBIC. Digestion was performed overnight at 37 °C. Reaction was stopped by adding 2% formic acid (FA) to the digestion solution. Gel pieces were washed and sonicated in 50% ACN/1% FA. Solution containing the resulting peptides were dried in SpeedVac, then redissolved and cleaned on C18 Spin Columns. Eluted samples were dried in SpeedVac and kept at – 20 °C. Samples were reconstituted in 25 µL 2% ACN, 0.1% FA before injection to a Waters Acquity I-Class UPLC system connected to Waters Select Series Cyclic IMS (Waters Corporation, Milford, UK). For peptide separation, samples were loaded on Waters Acquity Premier CSH column (150 × 1 mm, 1.7 µm) for multi-step gradient elution. Mobile phase (A) was composed of 0.1% FA in water, mobile phase (B) was composed of 0.1% FA in ACN. The elution method at flow rate 20 µL min^−1^ included the following: 1 min: 5% B, 45 min: 35% B, 46 min: 85% B at 45 °C. MS data acquisition was performed with the following parameters: m/z 50–2000, V-mode, scan time: 0.5 s, single Lock Mass: leucine enkephalin. MS^E^ fragmentation was performed in the trap: low energy: 6 V, high energy: ramping 19–45 V.

#### Data analysis

Data were analysed qualitatively using Waters ProteinLynx Global Server software. Parent and fragment ion tolerance was set to 20 ppm and 30 ppm respectively. Digestion enzyme was trypsin, missed cleavages were set to 2. Carbamidomethyl modification on cysteines was set as fixed modification, for variable modifications methionine oxidation was allowed. False discovery rate was 2%, and only proteins with minimal probability of 95% were counted. First, data were run in SwissProt database in order to collect contaminants. Then, analysis was performed in Maize and PSI-NDH databases (acquired form UniProtKB 03. 2021.), supplemented with the contaminant protein list. Processing parameters were the following: low energy threshold: 200 counts; elevated energy threshold: 20 counts; minimum fragment ion matches per peptide: 3; minimum fragment ion matches per protein: 7; minimum peptide matches per protein: 2.

### Statistics

Statistical analysis was performed using Prism v. 8.0.1 (GraphPad Software, Inc., San Diego, CA, United States). Methodology details are given in each figure legend. In general, data obtained from 2–3 separate isolations with (2)–3 technical replicates were compared except methodology data where only technical repetitions (3–6) were used to exclude differences among samples. Percentage data in Fig. 9a were compared using two-way ANOVA with Bonferroni’s multiple comparison test (*P* < 0.05) and in Additional file 1: Fig. S10 using one-way ANOVA with Tukey’s multiple comparison test (*P* < 0.05). Groups containing two members (Figs. 5g, 9a-inset, b, c) were compared with multiple t-tests (*P* < 0.05), and those with > 2 members (Figs. 3b, 7b, Additional file 1: Figs. S6 and S7) were compared using one-way ANOVA with Tukey’s multiple comparison test (*P* < 0.05). Comparisons were made separately in the different groups.

## Supplementary Information

**Additional file 1.** Additional figures S1–S12.

**Additional file 2.** BN-solubilisation and evaluation

**Additional file 3.** MS results 1.

**Additional file 4.** MS results 2.

**Additional file 5.** Gel preparations and buffers.

## Data Availability

All data generated or analysed during this study are included in this published article.

## Abbreviations

ACA: Aminocaproic acid
ACN: Acetonitrile
AMBIC: Ammonium bicarbonate
ATPs: ATP synthase
BN: Blue Native
BS: Bundle sheath
CF_1_: Coupling factor 1
Chl: Chlorophyll
CN: Clear Native
CP: Chlorophyll-protein
Cyt: Cytochrome
d: Dimer
*α*/*β*-DM: *n*-Dodecyl-*α*/*β*-d-maltoside
DOC: Sodium deoxychlolate
DS: *n*-Dodecyl-sucrose
EDTA: Ethylenediaminetetraacetic acid
FA: Formic acid
HEPES: 4-(2-Hydroxyethyl)-1-piperazineethanesulphonic acid
Lhc/LHC: Light-harvesting complex
LHCII-a: LHCII assembly complex containing CP29, CP24 and M-LHCII trimer
LM: Lincomycin
M: Mesophyll
m: Monomer
mc: Megacomplex
MS: Mass spectrometry
NDH: NADH dehydrogenase-like complex
PAGE: Polyacrylamide gel electrophoresis
PS: Photosystem
s: Supercomplex
SBG: Serva Blue G
SDS: Sodium dodecyl sulphate
t: Trimer
Tricine: *N*-(Tri(hydroxymethyl)methyl)glycine
Tris: Tris(hydroxymethyl)aminomethane
