# Supplementary material for: Qualitative and quantitative evaluation of thylakoid complexes separated by Blue Native PAGE
Source: Plant Methods. 2022 Mar 3;18:23. doi: 10.1186/s13007-022-00858-2 (PMC8895881; doi:10.1186/s13007-022-00858-2)
Supplement: Supplementary file 1 — Additional file 1. Additional figures S1–S12. [file 13007_2022_858_MOESM1_ESM.docx]

**
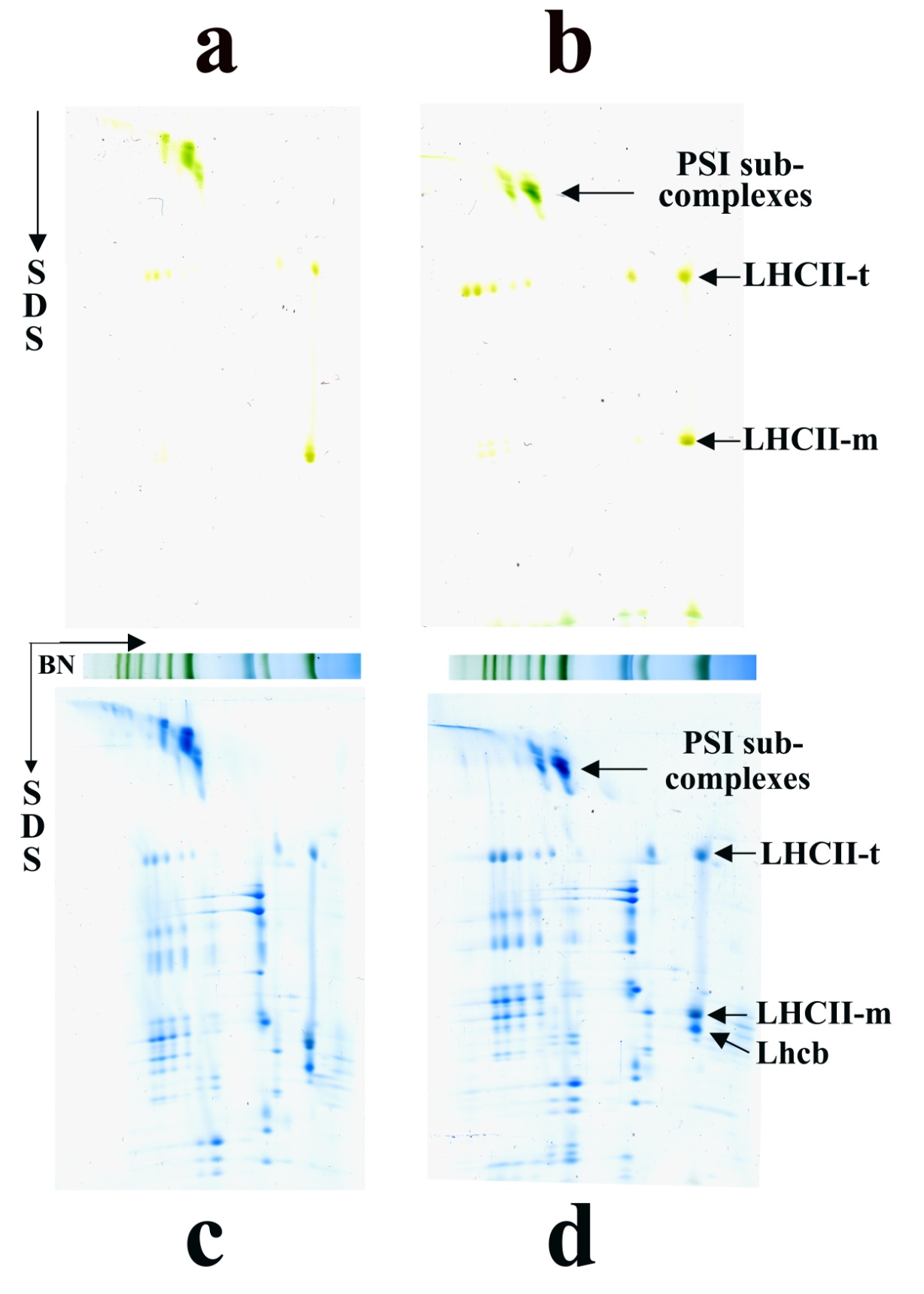
**

**Figure S1.** 2D BN/SDS PAGE patterns of maize mesophyll thylakoids. Thylakoids (500 µg Chl mL^-1^) were solubilised using 1% (w/V) *β*-DM plus 1% (w/V) digitonin, and separated in 4.3-12% gel gradient by BN PAGE followed by SDS PAGE. SDS gels without (**a**, **c**) or with (**b**, **d**) glycerol before (**a**, **b**) and after (**c**, **d**) Coomassie staining.

Spots are sharper in SDS gels containing glycerol. In addition, the native stage of large complexes was preserved better even under denaturing conditions in the presence of glycerol.

**
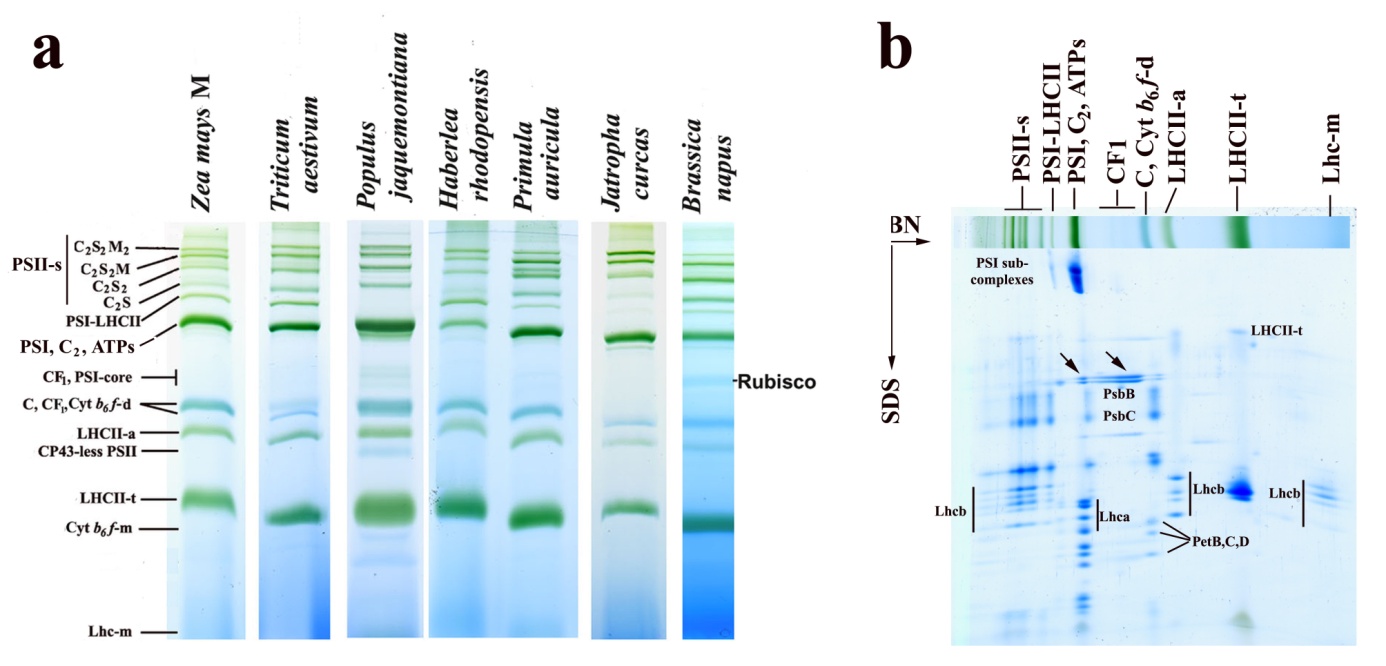
**

**Figure S2.** BN PAGE patterns and polypeptide profile of thylakoid complexes. (**a)** Complexes isolated from different plant species. Poplar thylakoids (500 µg Chl mL^-1^) were solubilised with 2% (w/V) *β*-DM, others with 1% (w/V) *β*-DM plus 1% (w/V) digitonin and separated in 5-12% BN gel gradient. M: mesophyll; m: monomer; d: dimer; t: trimer, s: supercomplex; LHCII-a: CP29 + CP24 + LHCII-t; Cyt: cytochrome; ATPs: ATP synthase; CF_1_: coupling factor 1. (**b)** BN/SDS PAGE pattern of *Primula auricula* thylakoid complexes.

**
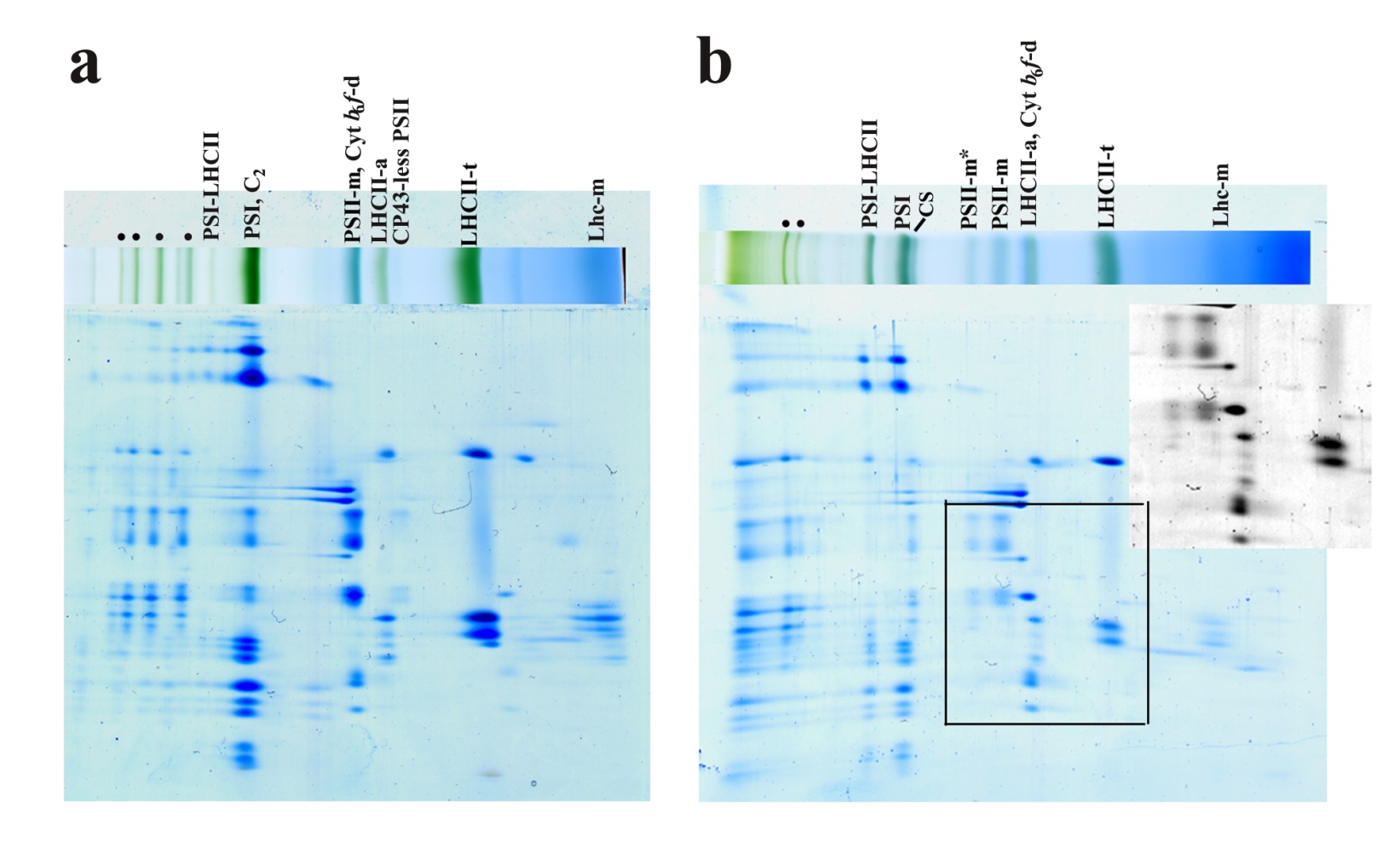
**

**Figure S3.** 2D BN/SDS PAGE of maize mesophyll thylakoids. Thylakoids (500 µg Chl mL^-1^) were solubilised using **a** 1% (w/V) *β*-DM plus 1% (w/V) digitonin or **b** 1% digitonin (inset: more contrasted pattern of the bordered part), and separated in 4.3-12% gel gradient by BN PAGE followed by SDS PAGE. Complexes are marked as in Figure S2a.

In digitonin-solubilised thylakoids, the band pattern showed the retention of the largest PSII supercomplexes and PSI-LHCII complex. Instead of PSII-d, a PSII complex running a little further than PSII-d and containing some LHCII (probably CS) was present in digitonin-solubilised samples. In addition, a somewhat larger monomer PSII (PSII-m*) band was also noticeable.


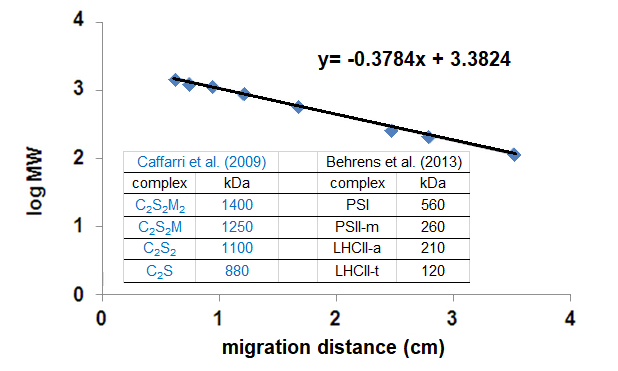


**Figure S4.** Calibration curve for molecular weight determination of complexes. Internal standards with molecular mass known from the literature [Caffarri et al. 2009; Behrens et al. 2013] were used for calibration. Maize thylakoids (500 µg Chl mL^-1^) were solubilised using 1% (w/V) *β-*DM plus 1% (w/V) digitonin and separated in 4.3–12% gel gradient by BN PAGE.


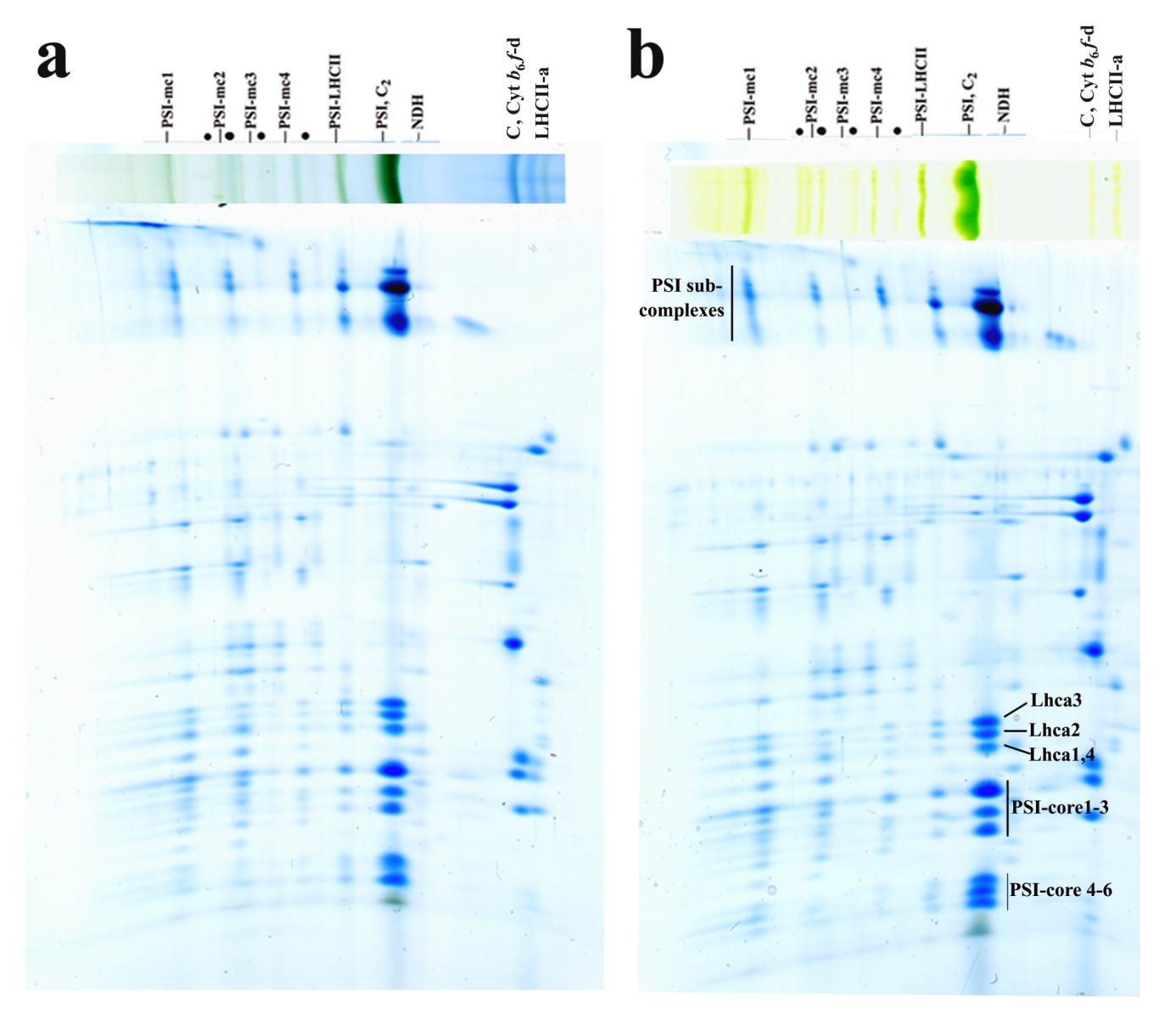


**Figure S5.** Polypeptide patterns of maize BS thylakoid preparations separated by **a** BN and **b** CN PAGE. Thylakoids (500 µg Chl mL^-1^) were solubilised using 1% (w/V) *β-*DM plus 1% (w/V) digitonin and were separated in 4.3-8% BN gel gradient followed by SDS PAGE. Complexes are marked as in Fig. 5d.

**
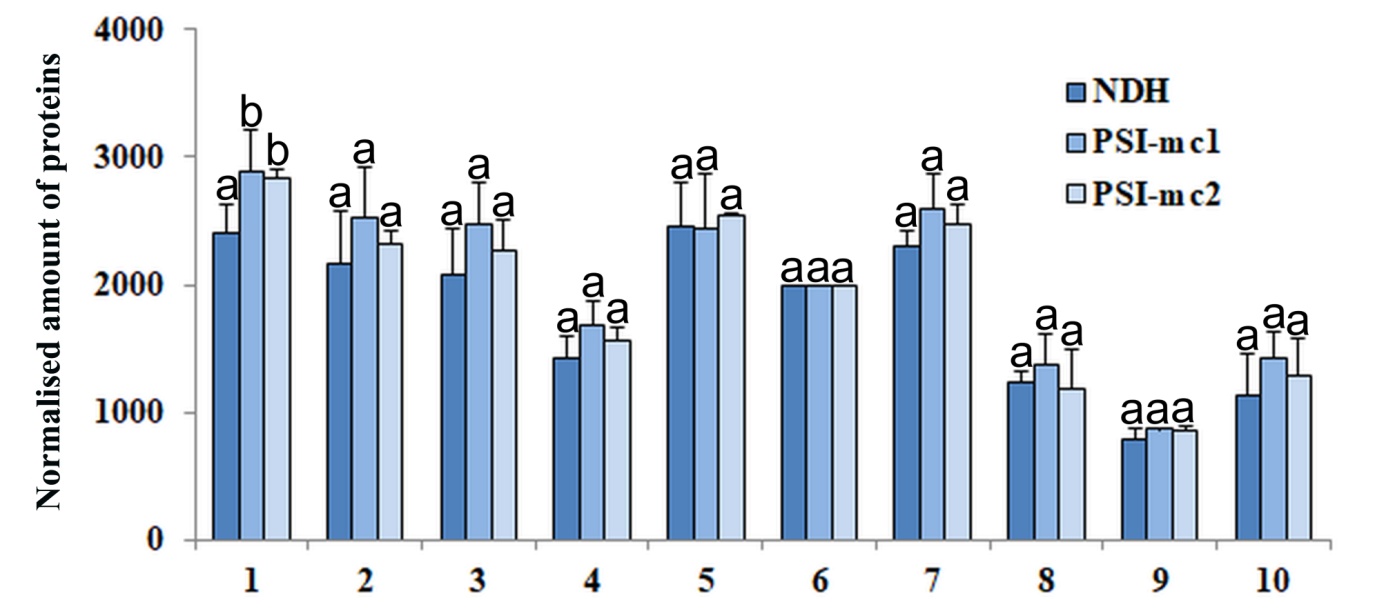
**

**Figure S6.** Comparison of the amounts of Ndh polypeptides normalised to spot 6 (marks as in Fig. 5c, f) in the NDH complex and PSI-NDH megacomplexes in maize BS thylakoids. The polypeptides were isolated by 3D PAGE as in Fig.5f: first-dimension BN (4.3-8%), second-dimension BN (4.3-12%) followed by SDS PAGE. Differences were compared using one-way ANOVA with Tukey’s multiple comparison test for each polypeptide group separately [*P*<0.05; n=3 × 2 (biological × technical)], and indicated by different letters.

**
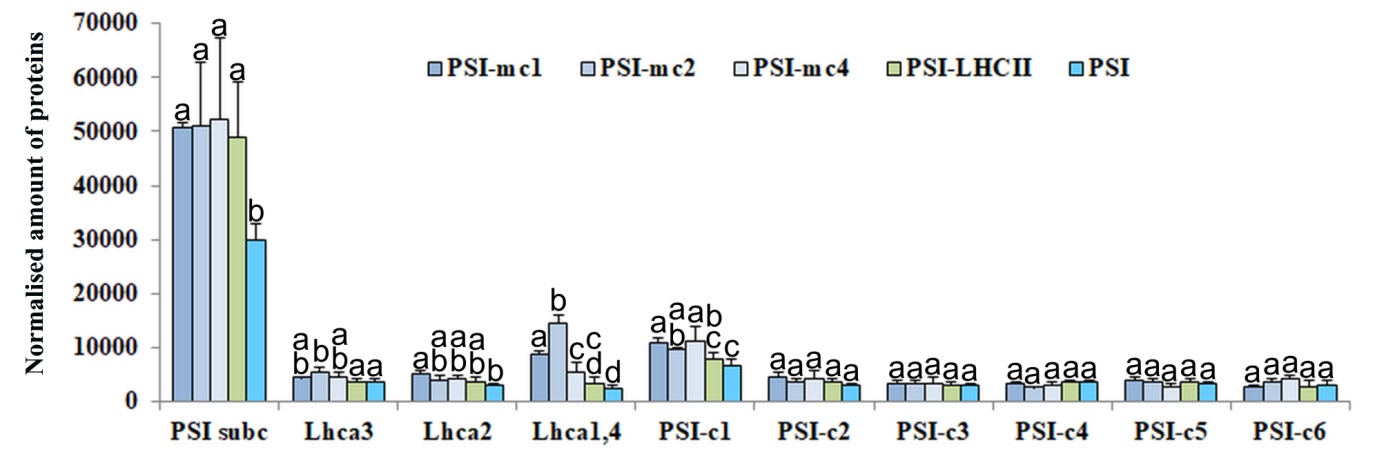
**

**Figure S7.** Comparison of the amount of the PSI polypeptides in the PSI complexes in maize BS thylakoids. Band volumes were normalised to the sum band volume of PSI core 4-6 (see Figure S5b for PSI polypeptides). Maize BS thylakoids (500 µg Chl mL^-1^) were solubilised using 1% (w/V) *β*-DM plus 1% (w/V) digitonin, and separated in 4.3–12% BN gel gradient followed by SDS PAGE. Differences were compared using one-way ANOVA with Tukey’s multiple comparison test for each polypeptide group separately [*P*<0.05; n=3 × 2 (biological × technical)], and indicated by different letters.

The low amount of PSI subcomplexes (PSI subc) in the PSI band is due to its very high density (cannot be measured precisely because of the too high absorbance). Higher variability of Lhca1,4 band and PSI-c1 are due to overlapping with Ndh polypeptides (see marks in Fig. 5c)*.*

**
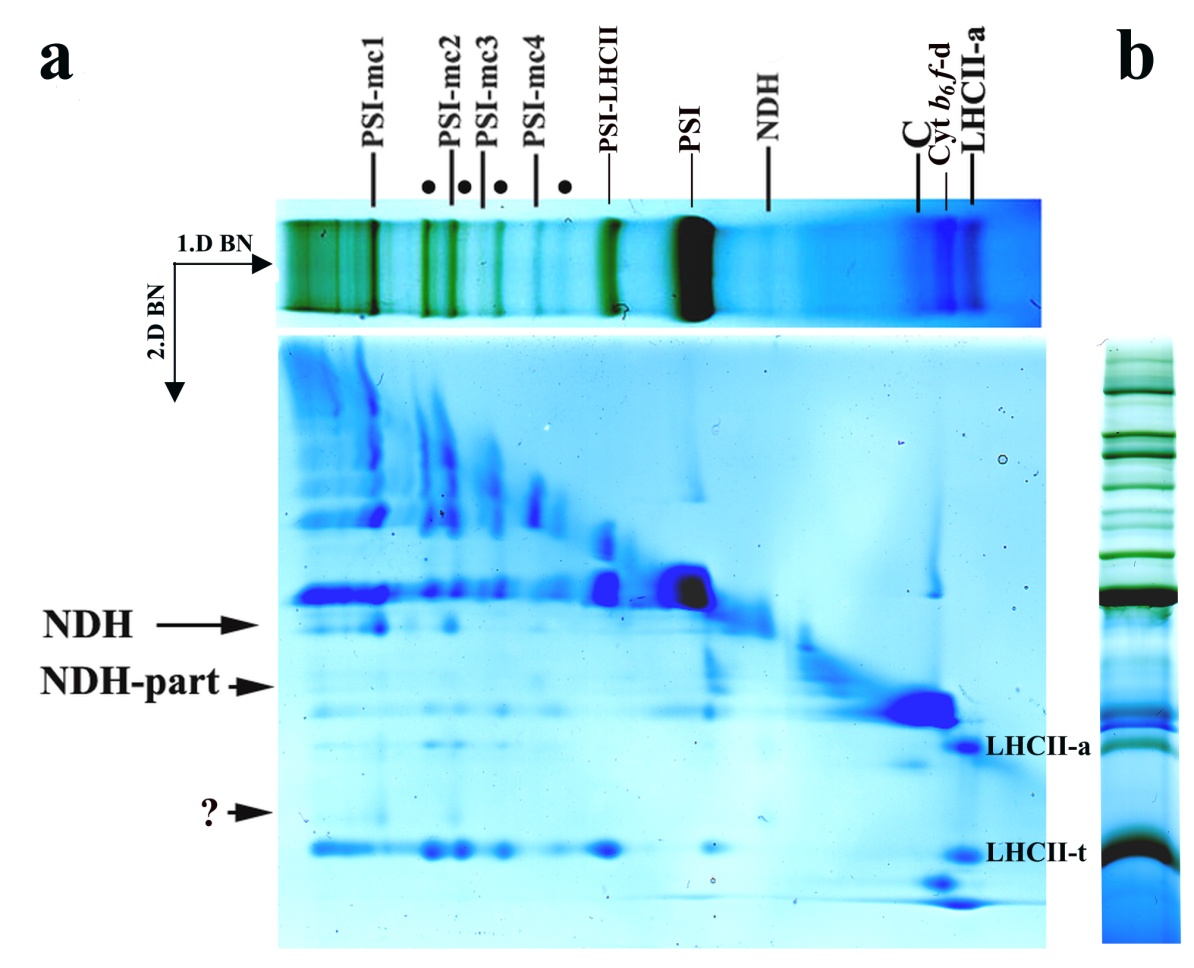
**

**Figure S8.** Two-dimensional BN patterns of maize BS thylakoids including bands used for mass spectrometry analyses. (**a**) Thylakoids (500 µg Chl mL^-1^) were solubilised using 1% (w/V) *β*-DM plus 1% (w/V) digitonin and separated in 4.3-8% BN gel gradient in the first dimension. Points show the PSII supercomplexes. Other complexes are marked as in Figure S5. In the second dimension the cut-out lanes were solubilised with 1% (w/V) *β*-DM and separated in 4.3–12% BN gel gradient. The second-dimensional pattern was stained with blue silver method. (**b**) BS thylakoids (500 µg Chl mL^-1^) were solubilised using 1% (w/V) *β-*DM plus 1% (w/V) digitonin and were separated in 4.3-12% BN gel gradient.

Different parts of the NDH complex seemed to be present in the two-dimensional BN pattern of BS thylakoids:

**NDH**: NDH complex without subcomplex B (see Table 1);

**NDH-part**: contains components of membrane and luminal subcomplexes of *Z.m*. (ndhA, pnsL1 and pnsL2, scores: 309^x^, 1015^x,y^ and 1946^x,y^, respectively);

**?**: It may be subcomplex B.


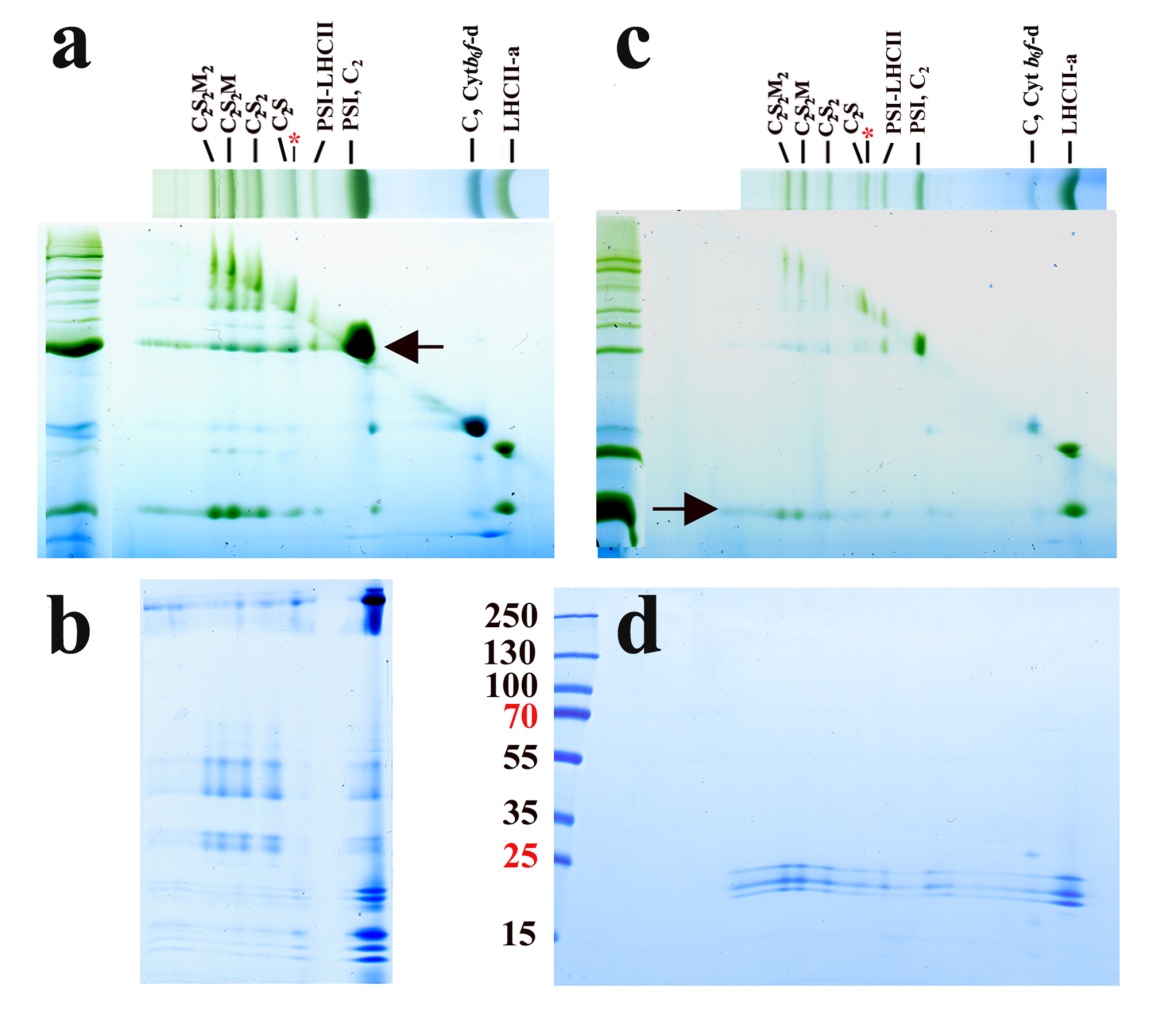


**Figure S9.** BN/BN/SDS PAGE of some maize thylakoid complexes. (**a), (c)** Two-dimensional BN PAGE of untreated (**a**) and lincomycin treated (**c**) mesophyll thylakoids. Thylakoids (500 µg Chl mL^-1^) were solubilised using 1% (w/V) *β*-DM plus 1% (w/V) digitonin, and separated in 4.3-8% gel gradient (first dimension), and after solubilisation with 1% *β*-DM in ice for 30 min and run in 4.3-12% gel gradient (second dimension). (**b), (d)** SDS PAGE of selected lanes marked with arrows (PSI zone in **a,** LHCII-t zone in **c**) on the BN/BN PAGE pattern.

In the PSI zone of the second-dimensional BN pattern (arrows in **a**), PSI monomers originating from PSI megacomplexes run together with C_2_, the main solubilisation product of the PSII supercomplexes after the release of some C, LHCII-a, and LHCII-t (Figure S9a). Near to the PSI band, a CS-like complex (Caffarri et al., 2009) can be also seen, which gives C and LHCII-t in second-dimension. Most oligomer bands above PSI seemed to contain Lhcb-s in lincomycin treated samples (Figure S9d).


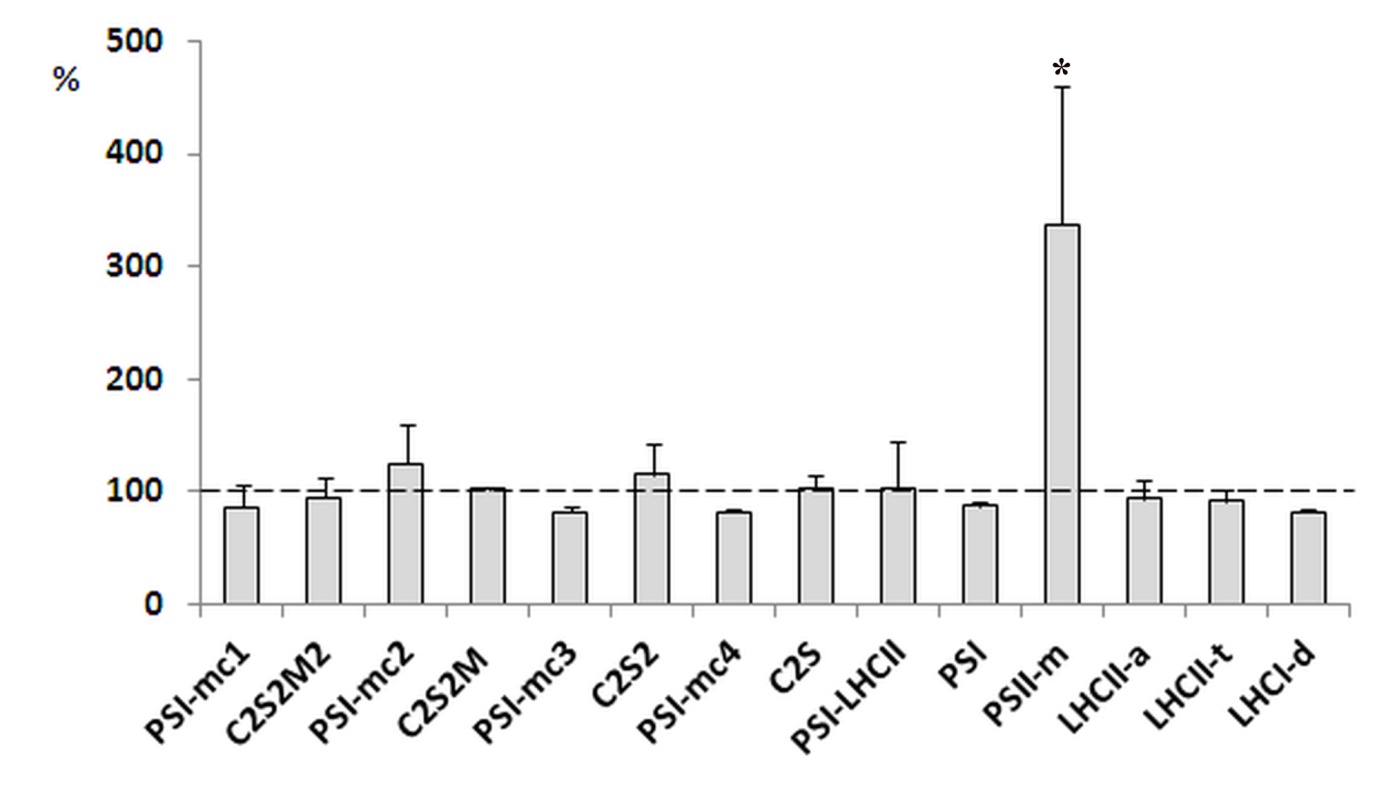


**Figure S10.** Comparison the band volumes of the complexes separated by CN- and BN PAGE. Maize untreated mesophyll, bundle sheath and lincomycin treated mesophyll thylakoids (500 µg Chl mL^-1^) were solubilised using 1% (w/V) *β*-DM plus 1% (w/V) digitonin and separated in 4.3-12% BN and CN gel gradient. In the different samples, band volumes of the same complexes were expressed as the percentage of that obtained in CN PAGE (100% line), and averaged. Differences were compared using one-way ANOVA with Tukey’s multiple comparison test [(*P*<0.05; n=3 × 2 (biological × technical)]. Significant differences are indicated by stars (*).


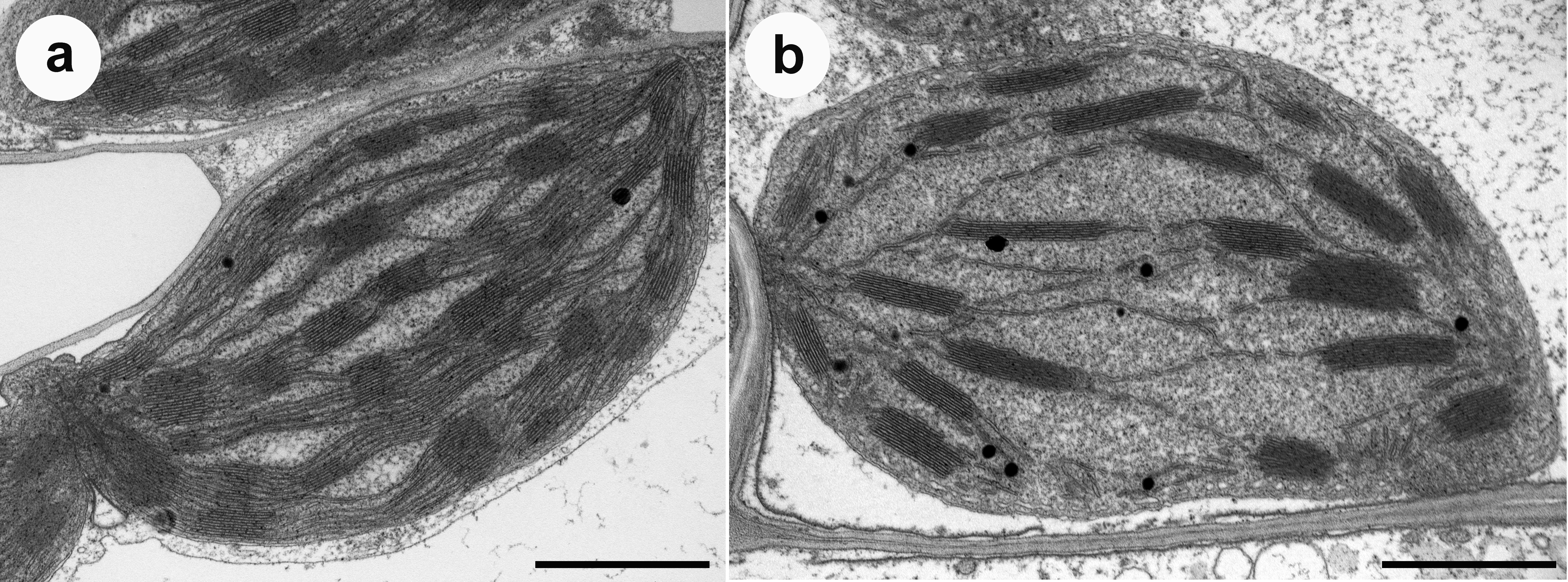


**Figure S10.** Transmission electron micrographs showing mesophyll chloroplast ultrastructure in (**a)** control and (**b)** lincomycin treated maize. Scale bar: 1 μm. Small, 1x1 mm maize leaf pieces were cut, then fixed in 2.5% glutaraldehyde and embedded in Durcupan resin as in Tóth et al. 2016. Ultrathin sections (70 nm) were cut using a Reichert-Jung ultramicrotome (Reichert-Jung AG, Austria), stained with Reynold’s lead citrate and uranyl-acetate and analysed by JEOL JEM 1011 (Jeol Ltd., Japan) transmission electron microscope with 80 kV accelerating voltage. Digital images were taken using an Olympus Morada CCD camera (Olympus Optical Co. Ltd., Japan).

Mesophyll chloroplasts had regular inner membrane structure with grana interconnected by stroma thylakoid membranes (**a**). Chloroplast inner membranes were strongly reduced in the lincomycin-treated maize leaves, with less grana and especially less developed stroma thylakoids (**b**). Similar mesophyll chloroplast structure has been reported in the literature [Sárvári et al. 1978, Tóth et al. 2016].


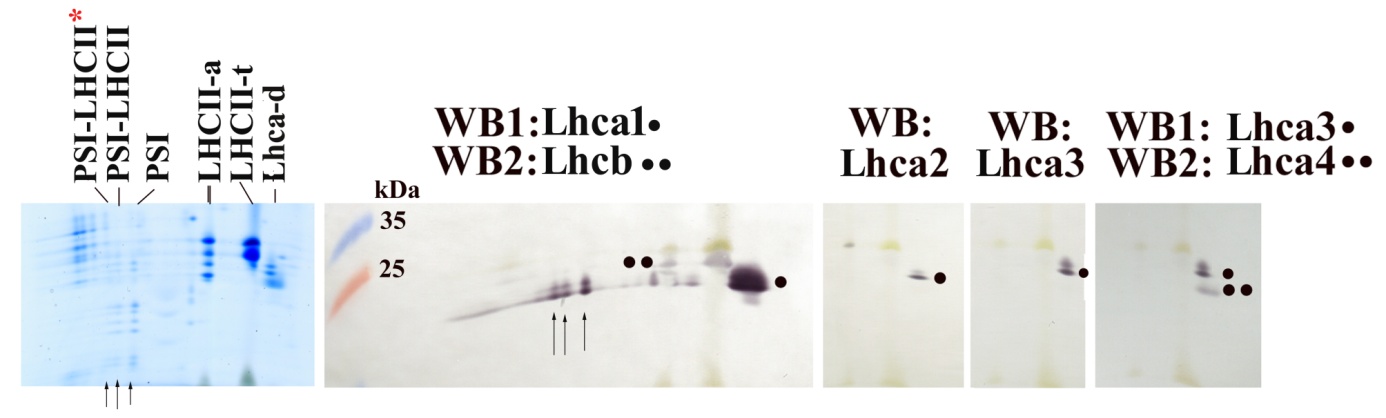


**Figure S12.** SDS PAGE and western blotting (WB) against Lhca and the main Lhcb polypeptides in mesophyll thylakoids isolated from lincomycin treated maize leaves. WB2: blotting was repeated on the same membrane by another antibody. Thylakoids (500 µg Chl mL^-1^) were solubilised using 1% (w/V) *β*-DM plus 1% (w/V) digitonin, and separated in 4.3–12% BN gel gradient followed by SDS PAGE and WB.

**References**

Behrens et al. 2013, DOI: 10.1016/j.jprot.2013.07.001

Caffarri et al. 2009, DOI: 10.138/emboj.2009.232

Sárvári et al. 1978, DOI: 10.1007/BF00387879

Tóth et al. 2016, DOI: 10.1016/j.bbabio.2016.04.287
