# Supplementary material for: Qualitative and quantitative evaluation of thylakoid complexes separated by Blue Native PAGE
Source: Plant Methods. 2022 Mar 3;18:23. doi: 10.1186/s13007-022-00858-2 (PMC8895881; doi:10.1186/s13007-022-00858-2)
Supplement: Supplementary file 5 — Additional file 5. Gel preparations and buffers. [file 13007_2022_858_MOESM5_ESM.docx]

**BN/CN PAGE**

**Solubilisation buffer:** 750 mM ACA, 50 mM BisTris-HCl, pH 7.0, 0.5 mM EDTA, 250 µg mL^-1^ Pefabloc and detergents. After solubilisation and centrifugation 100 µL supernatant was supplemented with 20 µL solution containing 750 mM ACA, 5% (w/V) SBG**.** For CN PAGE the supplement was 17.4% (w/V) glycerol.

**3xGel buffer**: 1.5 M ACA, 150 mM BisTris, pH 7.0 (adjusted with HCl)

**Cathode buffer:** 15 mM BisTris, 50 mM Tricine, pH 7.0 (*not adjusted*). CN PAGE: plus 0.02% (w/V) *β*-DM and 0.05% (w/V) DOC.

**Blue-cathode buffer:** cathode buffer containing 0.02% (w/V) SBG. *Store at room temperature!* 2^nd^D BN PAGE: plus 0.02% (w/V) *β*-DM.

**Anode buffer:** 50 mM BisTris, pH 7.0 (with HCl)

**AA/Bis solution:** 48% (w/V) acrylamide, 1.5% (w/V) bisacrylamide.

10% (w/V) **Ammonium persulfate (APS)**

**Gel casting** (in mL)**:** (for 8% gels 3 mL AA/Bis, and 4.45 mL DW was used in the higher concentration separating gel)

| BN/CN (mL) | 4 gels | 1.5 mm |  |
| --- | --- | --- | --- |
|  | Separating | | Stacking |
|  | 4.3% | 12.0% | 4.0% |
| 3xGel buffer | 6.0 | 6.0 | 6.0 |
| AA/Bis | 1.6 | 4.5 | 1.5 |
| 60% sucrose | 0.9 | 2.7 | - |
| 87% glycerol | 1.8 | 1.8 | 1.8 |
| DW | 7.8 | 2.95 | 8.45 |
| TEMED | 0.0085 | 0.0055 | 0.022 |
| 10% APS | 0.080 | 0.055 | 0.225 |
| sum volume | 18.0 | 18.0 | 18.0 |
|  |  |  |  |

Underlayered with 3 mL of 30% (w/V) sucrose (to fill tubes and groove under the gels)

Gels were usually made a day before use (they could be used for 1-3 days after casting casting packed in wet filter paper and plastic foil). Stacking gels are very soft and must be handled carefully.

**SDS PAGE**

**Solubilisation buffer**: 62.5 mM Tris-HCl (pH 6.8), 2% (w/V) SDS, 2% (w/V) dithiothreitol, 8.7% (w/V) glycerol, 0.001% (w/V) bromophenol blue (solubilisation of thylakoid samples: 1h at RT)

**AA/Bis solution:** 60% (w/V) AA, 1.6% (w/V) Bis

**Stacking gel**: 5% AA/Bis (w/V) (AA:BisAA=30:0.8), 0.125 M Tris-HCl (pH 6.8), 8.7% (w/V) glycerol, 0.1% (w/V) SDS, 0.056% (V/V) TEMED, 0.112% (w/V) APS

**Separating gel**: 10-18% (w/V) AA/Bis (AA:BisAA=30:0.8), 0.375 M Tris-HCl (pH 8.8), 8.7% (w/V) glycerol, 0.1% (w/V) SDS, 0.013-0.017% (V/V) TEMED gradient, 0.04% (w/V) APS, 3-9% (w/V) sucrose gradient

**Cathode/anode buffer**: 25 mM Tris, 192 mM glycine, 0.1% (w/V) SDS (pH 8.3)

| SDS gel casting (mL) | 8 gels | 1.5 mm |  |
| --- | --- | --- | --- |
|  | Separating | | Stacking |
|  | 10.0% | 18.0% | 5.0% |
| 4xgel buffer | 8.5 | 8.5 | 10.0 |
| AA/Bis | 5.7 | 10.2 | 3.3 |
| 60% sucrose | 1.7 | 5.1 | - |
| 87% glycerol | 3.4 | 3.4 | 4.0 |
| DW | 14.6 | 6.6 | 22.2 |
| TEMED | 0.0055 | 0.0045 | 0.022 |
| 10% APS | 0.128 | 0.128 | 0.440 |
| sum volume | 34.0 | 34.0 | 40.0 |

underlayered with 3 mL of 30% (w/V) sucrose

Gels were usually made a day before use (they could be used after casting packed in wet filter paper and plastic foil for a week).
